# Thermo diffusion impacts on the flow of second grade fluid with application of (ABC), (CF) and (CPC) subject to exponential heating

**DOI:** 10.1038/s41598-022-21773-5

**Published:** 2022-11-02

**Authors:** Aziz Ur Rehman, Muhammad Bilal Riaz, Adam Wojciechowski

**Affiliations:** 1 Department of Mathematics, University of Management and Technology, 54770 Lahore, Pakistan; 2 Faculty of Technical Physics, Information Technology and Applied Mathematics, Lodz University of Technology, 90-924 Lodz, Poland

**Keywords:** Engineering, Mathematics and computing, Physics

## Abstract

The aim of this article is to investigate the exact solution by using a new approach for the thermal transport phenomena of second grade fluid flow under the impact of MHD along with exponential heating as well as Darcy’s law. The phenomenon has been expressed in terms of partial differential equations, then transformed the governing equations in non-dimentional form. For the sake of better rheology of second grade fluid, developed a fractional model by applying the new definition of Constant Proportional-Caputo hybrid derivative (CPC), Atangana Baleanu in Caputo sense (ABC) and Caputo Fabrizio (CF) fractional derivative operators that describe the generalized memory effects. For seeking exact solutions in terms of Mittag-Leffler and G-functions for velocity, temperature and concentration equations, Laplace integral transformation technique is applied. For physical significance of various system parameters on fluid velocity, concentration and temperature distributions are demonstrated through various graphs by using graphical software. Furthermore, for being validated the acquired solutions, accomplished a comparative analysis with some published work. It is also analyzed that for exponential heating and non-uniform velocity conditions, the CPC fractional operator is the finest fractional model to describe the memory effect of velocity, energy and concentration profile. Moreover, the graphical representations of the analytical solutions illustrated the main results of the present work. Also, in the literature, it is observed that to derived analytical results from fractional fluid models developed by the various fractional operators, is difficult and this article contributing to answer the open problem of obtaining analytical solutions the fractionalized fluid models.

## Introduction

The process of heat and mass transfer has a great importance from the industrial point of view. Many researchers and scientists concentrate on this area. In modern technologies and various industrial fields, the non-Newtonian fluid theory has extensive impact because Newtonian fluid model can not express many flow characteristics. A non-Newtonian fluid that obeys the nonlinear relationships between the rate of shear strain and the shear stress. The non-Newtonian fluid theory has significant utilization in modern engineering, especially in petroleum industry used to extract crude oil from different petroleum productions. The properties of Newtonian fluid in most of the cases are not valid but scientists desire to model the complex models for non-Newtonian fluid. The importance of non-Newtonian fluid has been enlarged from the last few decades, specifically in the research field. The non-Newtonian fluids have numerous ever-increasing applications in industrial sectors, but some specific are mentioned here, such as at large-scales reducing and enhancing heating/cooling systems, biochemical and process engineering, extrusion of molten plastic in industry, reducing oil pipeline friction, polymer processing, reducing fluid friction, well drilling, flow tracers, biological materials, biomedical flow analysis, plastic foam processing, lubrication processes, food processing industries, chemical processing, all emulsions, handling of muds, slurries and complex mixtures. Many researchers and scientists focused on non-Newtonian fluid while considering different fluid geometries. Therefore simulating and modelling the flow phenomena of non-Newtonian fluid that is facilitated and play the important role in human life. Researchers investigated different non-Newtonian fluid models regarding physical and computational characteristics such as second grade model, viscoplastic model, power law model, Bingham plastic model, Jeffery model, Oldroyd-B fluid model, Brinkman type model, Casson model, Walters-B fluid model and Maxwell model^1–5^, that different fluid models exists in the literature have various characteristics or certain limitations, for instance, the power-law model described the features of viscosity but failed to explain the impacts of elasticity, which motivate/attract the researchers and mathematicians towards the study of such complex fluids. Multiple products such as honey, soup, jelly, china clay, tomato sauce, artificial fibers, synthetic lubricants, concentrated fruit juices, pharmaceutical chemicals, paints and coal, etc. are some applied illustrations of such fluid. Systematic analysis of such fluid flow models have significantly important for theoretical studies and practical implementations in modernistic mechanization. Among such fluids, second grade fluid attracted special attention, which is the commonest non-Newtonian fluid due to its more extensive applications and substantial role in different fields serving as mechanical as well as chemical applications, bio engineering operations, metallurgy and especially in food processing industries. Lubricants that are used to lubricate the components of engine like gears, bearings, etc., are considered as differential type fluids. The study of second grade movement in the context of fluid mechanics, was explored by several mathematicians, scientists, researchers and engineers that depends upon various situations. Rajagopal et al.^6,7^ examined the influence of various applications of differential type fluid, for instance, in theological problems, in biological sciences, chemical, petroleum and geophysics field. The parabolic partial differential equations governing the flow in the presence of a magnetic field, Hall currents and the free stream velocity has been studied by Takhar et al.^8^ Modather et al.^9^ presented the analytical solution of the problem related to MHD mass and heat transfer of an oscillatory two-dimensional viscous fluid that is electrically conducting over an infinite vertical permeable moving plate which is embedded in a porous medium along with a chemical reaction and transverse magnetic field.

Viscosity of fluid keeps its leading role in the biological fluids, polymer process, mayonnaise, melt solutions, colloidal suspensions and lubrication models. The Carreau nanofluid viscosity model can explain features of non-Newtonian fluids in the shear-thinning/thickening regions. Carreau viscosity model and thermal radiation under the influence of non-uniform heat source/sink transportation phenomenon of heat over the surface was discussed by Assad et al.^10^ and Ali et al.^11^ analyzed the characteristics of upper-convected second grade nanofluid thin film flow over a time-dependent stretching sheet with variable thermal conductivity and Cattaneo–Christov double diffusion theory.

Some researchers and scientists are focused to investigate the flow regime of second grade fluid geometrically for configurations of many interesting features, because flow analysis of differential type fluids have wide practical applications and theoretically studies, having prominent effects in many industrial fields, for example, Erdogan^12^, Labropulu^13^, Fetecau et al.^14^, Tawari and Ravi^15^ and Islam et al.^16^ studied the unsteady second grade fluid, employed the method of separation of variables, to compute analytical solution. Rehman et al.^17^ elaborated the MHD second grade fluid flow to analyzed the effects of radiative thermal flux and computed the analytical solutions by employing Laplace integral transformation. Ali^18^ investigated free convection flow of an electrically conducting fluid along a vertical plate embedded in a thermally stratified porous medium in the presence of a uniform normal magnetic field. MHD mixed convection from a semi-infinite, isothermal, vertical and permeable surface immersed in a uniform porous medium in the presence of thermal radiation and Dufour and Soret effects are studied by Ali^19^. Assad et al.^20^ presented a comprehensive analysis of the second-order velocity slip phenomenon and stagnation point flow of cross nanofluid with a spectral relaxation approach over the geometry of porous medium. Some significant studies regarding second grade fluid having interesting facts are described by Rashidi et al.^21^, Baranovskii^22^, Arianna et al.^23^, Dinarvand et al.^24^ and Fetecau et al.^25^

The fractional/differential calculus is an eminent mathematical field that growing immensely due to enormous significance investigates the non integer order behavior of integrals and derivatives as well as their applications and properties. The concept of differential calculus is old like classical calculus, first time in 1695, new idea about fractional calculus introduced when a letter from Leibniz to L’Hospital was written. This field attracted the attention of well known mathematicians, researchers and scientists that proposed and built different fractional integrals and fractional derivatives. The researchers faced too much difficulties to developed a real physical phenomenon by employing the traditional calculus techniques, then the fractional differential equations have great importance for mathematicians and researchers. Since it has been investigating numerous physical models in different scientific fields such as biology, physics, chemistry, acoustic waves, finance, control theory, fractal dynamics, signal processing, hydro magnetic waves, diffusion reaction process, anomalous transport, fluid flow problems, engineering processes, oscillation, dynamical processes and many other disciplines. The main reason for exploring the numerical or exact solutions due to its significance in various daily life. To gain the numerical or exact solutions, researchers and mathematicians have been implemented numerous techniques. For instance, unified method^26^, multi step approach^27,28^, Riccati-Bernouli sub-ordinary diffrential equation Sub-ODE techniq (RBSODET)^29^,reproducing the kernel Hilbert space method^30,31^, simple equation modification method^32^, residual power series method^33^ and several others^34–36^. Mehmet et al.^37^ applied $\rho$-Laplace homotopy transform method ($\rho$-LHTM) and the heat balance integral method (HBIM) to solve the fractional incompressible second-grade fluid differential equations. Due to the advancement in the field of fractional calculus, scientists have suggested a couple of new techniques to interpret and established the real world problem solutions using theory of fractional calculus. To interpret and model phenomenon in different fields of sciences such as electric circuit models, fractal rheological models and fractal growth of populations models, several fractional operators have singular kernels but a lot of having non-singular kernels have been acquired, which is an important tool to analyze the rheological behavior of the physical models in fractional calculus. In literature, many researchers surprisingly work a lot in this shining field of mathematics to analyzed the fractional fluid models and derived various interesting results that are very helpful for engineers and scientists to compare their experimental results get from the govern partial differential equations with the analytical results obtained using different mathematical techniques and tools from fractional form of the non-Newtonian fluid models. Marchaud Caputo and Riemann–Liouville developed fractional integrals and described a new concept of fractional derivatives operators, that are based on singular kernels, but these fractional models have some drawbacks due to the singular kernels such as faced many difficulties during modeling process. To overcome this hurdle that occurred singularized fractional models , a new set of fractional operators have been presented that are based on non-singular kernels, such as Prabhakar fractional derivative, Caputo-Fabrizio, Yang Abdel Cattani fractional, Atangana–Baleanu fractional operators and few others for reference^38–43^. These fractional operators having different type of non-singularized kernels, some of the kernels are mentioned here such as, Rabotnov exponential function, Exponential kernels and Mittag-Leffler functions. Mehmet et al.^44^ investigated the exact solution and a qualitative study for the fractional second-grade fluid described by a Caputo fractional operator. Aziz et al.^45^ has been discussed heat source impact on unsteady magneto-hydro-dynamic (MHD) flows of Prabhakar-like non integer second grade fluid near an exponentially accelerated vertical plate with exponentially variable velocity, temperature and mass diffusion through a porous medium. The analytical treatment to the bio heat transfer Pennes model via modern fractional derivatives are analyzed by Kashif et al.^46^ Comprehensive analysis of heat and mass transfer of MHD natural convection flow of water-based nano-particles in the presence of ramped conditions via Caputo-Fabrizio fractional time derivative are investigated by Aziz et al.^47^

In the previous investigation, Sami Ul Haq et al.^48^ and Ying-Qing Song et al.^49^ discussed the flow of fractional version of differential type fluid model by using different fractional operators namely CF and ABC, respectively, and computed solution for each fractional model, but both respective studies presented work without analyzed the effect of mass diffusion. But in the literature fractional second grade fluid model with fractional operators CPC, CF and ABC, along with the set of non-uniform boundary conditions for velocity with exponential heating, saturated in porous media, are not investigated yet nor published. To fill this gape a new fractional second grade fluid model developed by applying the definition of recently introduced a new fractional derivative operator, namely CPC, CF and ABC operators, under effectively applied conditions for concentration, velocity field and temperature distribution. For seeking exact solution expressions in terms of G-functions by employing Laplace integral transformation method to solve the fractional models developed for velocity, concentration and temperature distribution. For physical analysis the influence of parameters like as second grade parameter $\alpha _2$, dimensionless time *t*, fractional parameter $\alpha$, mass Grashof number *Gm*, Magnetic number *M*, Prandtl number *Pr*, Schmidt number *Sc*, thermal Grashof number *Gr* are portrayed graphically by using Mathcad software. Furthermore, for validation the current result, also accomplish the comparative analysis with different published work.

## Mathematical model

**Figure 1:**
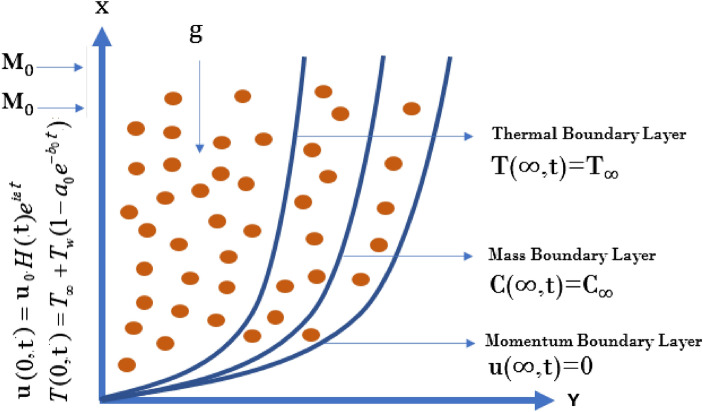
Physical geometry of the second grade fluid model.

Consider the MHD second grade fluid flow near an oscillating infinite vertical plate that is nested in a porous material. The plate is considered at $y= 0$ and the fluid flow is restrained to $y > 0$, in thedirection that is along to the plate (as exhibited in Fig. 1). Initially, for time $t ={0}$, the fluid and plate both are in the static mode, having ambient temperature ${{T}_{\infty }}$ and concentration ${{C}_{\infty }}$. Later, when time $t ={{0}^{+}}$, the plate begins to oscillate and fluid starts to move with velocity $u_{0}H(t)e^{i \epsilon t}$, where $\epsilon$ represents oscillation frequency, *H*(*t*) represented unit step function and $u_{0}$ represents characteristic velocity, and the wall temperature is $T_w$ and concentration $C(0,t)=C_w$. It is presumed that temperature, velocity and concentrations are functions of *y* and *t* only.The following principal equations for second grade fluid under Boussinesq’s approximation, for velocity,concentration and energy transfer are obtained as^50,51^:

1 $$\begin{aligned} \frac{\partial {u(y,t)}}{\partial {t}}&= \upsilon \left( 1+\frac{\alpha _1}{\mu }\frac{\partial }{\partial {t}}\right) \frac{\partial ^{2}{u(y,t)}}{\partial {y}^{2}} +g\beta _T\left( T(y,t)-T_{\infty }\right) +g\beta _{C}\left( C(y,t)-C_{\infty }\right) \nonumber \\&\quad -\left[ \frac{{\sigma _{0}}M_{0}^{2}}{\rho }+\frac{\upsilon \phi }{k_{0}}\left( 1+\frac{\alpha _1}{\mu }\frac{\partial }{\partial {t}}\right) \right] u(y,t), \end{aligned}$$

2 $$\begin{aligned}&\frac{\partial {T(y,t)}}{\partial {t}}=\frac{k}{{\rho }{{C}_{p}}}\frac{\partial ^{2}{T(y,t)}}{\partial {y}^{2}}, \end{aligned}$$

3 $$\begin{aligned}&\frac{\partial {C(y,t)}}{\partial {t}}=\delta _{m}\frac{\partial ^{2}{C(y,t)}}{\partial {y}^{2}}- \delta _{m}\left( C(y,t)-C_{\infty }\right) , \end{aligned}$$

where $u(y,t), T(y,t), C(y,t), \rho , \beta _T, \alpha _1, k$, $\upsilon$, $C_p$ , $\beta _C$ and *g* represents the fluid velocity, temperature, concentration, density, coefficient of volumetric thermal expansion, second grade parameter, thermal conductivity, kinematic viscosity, specific heat capacity, coefficient of volumetric expansion for concentration and gravitational acceleration respectively.

With initial (ICs) and boundary (BCs) conditions are given below:

4 $$\begin{aligned}&u(y,0)=0, \quad T(y,0)=T_{\infty },\quad C(y,0)=C_{\infty }, \quad \frac{\partial {u(y,t)}}{\partial {t}}=0, \quad y \ge 0, \end{aligned}$$

5 $$\begin{aligned}&u(0,t)=u_{0}H(t)e^{i \epsilon t},\quad T(0,t)=T_{\infty } + T_{w}(1-a_0e^{-b_0 t}),\quad C(0,t)=C_{w} \quad t >0 ,u_0 \ne 0, \end{aligned}$$

6 $$\begin{aligned}&u(y,t) \rightarrow 0, \quad T(y,t) \rightarrow T_{\infty },~~C(y,t)\rightarrow C_{\infty } ~~as~~y \rightarrow \infty . \end{aligned}$$

Writing the proposed problem in terms of dimensionless form, the following dimensionless quantities are considered:

7 $$\begin{aligned} u^{*}&=\frac{{u}}{u_0}, \quad y ^*=\frac{u_0}{\upsilon }y,\quad t^*=\frac{u_0^2}{\upsilon }t, \quad \theta =\frac{T-T_{\infty }}{T_{w}-T_{\infty }},\quad G_{r}=\frac{g \upsilon \beta _{\grave{T}}(T-T_{\infty })}{u_{0}^3},\nonumber \\ G_{m}&=\frac{g\upsilon \beta _{C}(C-C_{\infty })}{u_{0}^3},\quad M=\frac{{\sigma _{0}}M_{0}^{2}\upsilon }{{\rho }u_0^2},\quad Pr=\frac{{\mu }{C_{p}}}{k}, \quad Sc=\frac{\upsilon }{\delta _{m}},\quad \lambda =\frac{\upsilon ^2}{u_0^2},\nonumber \\ C^{*}&=\frac{C-C_{\infty }}{C_{w}-C_{\infty }},\quad \frac{1}{K}=\frac{{\upsilon ^2}\phi }{k_0{u_0^2}},\quad \alpha _2=\frac{{\alpha _1}{\rho }{u_0^2}}{\mu ^2},\quad a=M+\frac{1}{K},\quad b=\frac{\alpha _2}{K}. \end{aligned}$$

When substituting the Eq. (7) into Eqs. (1), (2) and (3), and dropping the asterisk $*$ from newly obtained equations, then we have the dimensionless governing system of PDEs of the considered model as follows:

8 $$\begin{aligned} \frac{\partial {u(y,t)}}{\partial {t}}=\frac{\partial ^{2}{u(y,t)}}{\partial {y}^{2}} +G_{r}\theta (y,t)+G_{m} C(y,t)-au(y,t)-b\frac{\partial {u(y,t)}}{\partial {t}}+ \alpha _2 \frac{\partial ^{3}{u(y,t)}}{{\partial {t}}\partial {y^2}}, \end{aligned}$$

9 $$\begin{aligned}&\frac{\partial {\theta (y,t)}}{\partial {t}}=\frac{1}{Pr}\frac{\partial ^{2}{\theta (y,t)}}{\partial {y}^{2}}, \end{aligned}$$

10 $$\begin{aligned}&\frac{\partial {C(y,t)}}{\partial {t}}=\frac{1}{Sc}\frac{\partial ^{2}{C(y,t)}}{\partial {y}^{2}}-\frac{\lambda }{Sc}C(y,t). \end{aligned}$$

with

11 $$\begin{aligned}&u(y,0)=0, \quad \theta (y,0)=0, \quad C(y,0)=0, \end{aligned}$$

12 $$\begin{aligned}&u(0,t)=H(t)e^{i \epsilon t},\quad \theta (0,t)=1-a_0 e^{-b_0 t},\quad C(0,t)=1 , \quad t >0, \end{aligned}$$

13 $$\begin{aligned} u(y,t) \rightarrow 0, \quad \theta (y,t) \rightarrow 0,~~C(y,t)\rightarrow 0 ~~as~~y \rightarrow \infty . \end{aligned}$$

The fractional model for momentum, energy and concentration distribution are formulated by using Constant proportional-Caputo hybrid fractional derivative operator is described as:

14 $$\begin{aligned}&{^{CPC}D_{t}^{\alpha }} u(y,t) = \left( 1 + \alpha _2 {^{CPC}D_{t}^{\alpha }}\right) \frac{\partial ^{2}{u(y,t)}}{\partial {y}^{2}} +G_{r}\theta (y,t)+G_{m} C(y,t)-(a + b{^{CPC}D_{t}^{\alpha }})u(y,t), \end{aligned}$$

15 $$\begin{aligned}&{^{CPC}D_{t}^{\alpha }} \theta (y,t)=\frac{1}{Pr}\frac{\partial ^{2}{\theta (y,t)}}{\partial {y}^{2}}, \end{aligned}$$

16 $$\begin{aligned}&{^{CPC}D_{t}^{\alpha }} C (y,t) =\frac{1}{Sc}\frac{\partial ^{2}{C(y,t)}}{\partial {y}^{2}}-\frac{\lambda }{Sc}C(y,t). \end{aligned}$$

The fractional model for momentum, energy and concentration distribution are formulated by using Caputo–Fabrizio fractional derivative operator is described as:

17 $$\begin{aligned} {^{CF}D_{t}^{\alpha }} u(y,t)&= \left( 1 + \alpha _2 {^{CF}D_{t}^{\alpha }}\right) \frac{\partial ^{2}{u(y,t)}}{\partial {y}^{2}} +G_{r}\theta (y,t)+G_{m} C(y,t)-(a + b{^{CF}D_{t}^{\alpha }})u(y,t), \end{aligned}$$

18 $$\begin{aligned} {^{CF}D_{t}^{\alpha }} \theta (y,t)&=\frac{1}{Pr}\frac{\partial ^{2}{\theta (y,t)}}{\partial {y}^{2}}, \end{aligned}$$

19 $$\begin{aligned} {^{CF}D_{t}^{\alpha }} C (y,t)&=\frac{1}{Sc}\frac{\partial ^{2}{C(y,t)}}{\partial {y}^{2}}-\frac{\lambda }{Sc}C(y,t). \end{aligned}$$

Also,The fractional model for momentum, energy and concentration distribution are formulated by using Atangana–Baleanu time fractional operator is described as:

20 $$\begin{aligned} {^{ABC}D_{t}^{\alpha }} u(y,t)&= \left( 1 + \alpha _2 {^{ABC}D_{t}^{\alpha }}\right) \frac{\partial ^{2}{u(y,t)}}{\partial {y}^{2}} +G_{r}\theta (y,t)+G_{m} C(y,t)-(a + b{^{ABC}D_{t}^{\alpha }})u(y,t), \end{aligned}$$

21 $$\begin{aligned} {^{ABC}D_{t}^{\alpha }} \theta (y,t)&=\frac{1}{Pr}\frac{\partial ^{2}{\theta (y,t)}}{\partial {y}^{2}}, \end{aligned}$$

22 $$\begin{aligned} {^{ABC}D_{t}^{\alpha }} C (y,t)&=\frac{1}{Sc}\frac{\partial ^{2}{C(y,t)}}{\partial {y}^{2}}-\frac{\lambda }{Sc}C(y,t), \end{aligned}$$

where, ${^{CPC}D_{t}^{\alpha }} (.,.)$ represents Constant proportional-Caputo hybrid fractional operator and its defined as:

$$\begin{aligned} ^{CPC}D_{t}^{\alpha }f\left( y,t\right) =\frac{1}{\Gamma (1-\alpha )}\int _{0}^{t} (k_{1}(\alpha )f(y,\tau )+k_{0}(\alpha )\frac{\partial f(y,\tau )}{\partial \tau })(t-\tau )^{-\alpha }d\tau ,\quad 0<\alpha <1. \end{aligned}$$

Laplace transformation of Constant proportional-Caputo hybrid time fractional operator is written as:

$$\begin{aligned} \mathcal L\left( {^{CPC}D_{t}^{\alpha }f\left( y,t\right) }\right) =\left[ \frac{k_{1}(\alpha )}{s} + k_{0}(\alpha )\right] s^{\alpha } \mathcal L\left( f(y,t)\right) -k_{0}(\alpha )s^{\alpha -1}f(y,0), \end{aligned}$$

where $\alpha$ represents a fractional parameter.

Now, ${^{CF}D_{t}^{\alpha }} (.,.)$ represents Caputo–Fabrizio fractional operator and its defined as:

$$\begin{aligned} ^{CF}D_{t}^{\alpha }f\left( y,t\right) =\frac{1}{1-\alpha }\int _{0}^{t} exp \left( -\frac{\alpha (t-\alpha )}{1-\alpha }\right) \frac{\partial f(y,\tau )}{\partial \tau }d\tau ,\quad 0<\alpha <1. \end{aligned}$$

Laplace transformation of Caputo–Fabrizio time fractional operator is written as:

$$\begin{aligned} \mathcal L\left( {^{CF}D_{t}^{\alpha }f\left( y,t\right) }\right) = \frac{s \mathcal L\left( f(y,t)\right) -f(y,0)}{(1-\alpha )s+\alpha }, \end{aligned}$$

where $\alpha$ represents a fractional parameter.

Also, ${^{ABC}D_{t}^{\alpha }} (.,.)$ represents Atangana–Baleanu time fractional operator in Caputo sense (ABC) having non-singularized and non-local kernel defined in the following way:

$$\begin{aligned} ^{ABC}D_{t}^{\alpha }f\left( y,t\right) = \frac{1}{1-\alpha }\int _{0}^{t} E_{\alpha } \left( -\frac{\alpha (t-\alpha )}{1-\alpha }\right) \frac{\partial f(y,\tau )}{\partial \tau }d\tau ,\quad 0<\alpha <1 \end{aligned}$$

its Laplace transformation is obtained as:

$$\begin{aligned} \mathcal L\left( {^{ABC}D_{t}^{\alpha }f\left( y,t\right) }\right) =\frac{s^{\alpha } \mathcal L\left( f(y,t)\right) -s^{\alpha -1}f(y,0)}{(1-\alpha )s^{\alpha }+\alpha }, \end{aligned}$$

where $\alpha$ is named as fractional parameter.

## Solution of the flow problem

In this section, the analytical solution derived of the non-dimensional fractionalized second grade fluid model by employing the technique of Laplace transformation.

### Temperature equation solution by using CPC derivative operator

Employing Laplace integral transformation to Eq. (15) with conditions given in Eqs. (11, 12, 13), we have

23 $$\begin{aligned} \frac{d^{2}{\bar{ \theta }(y,s)}}{d{y}^{2}}-P_r \left[ \frac{k_{1}(\alpha )}{s} + k_{0}(\alpha )\right] s^{\alpha }{\bar{\theta }(y,s)}=0, \end{aligned}$$

with

24 $$\begin{aligned} {\bar{\theta }(0,s)}=\frac{1}{s}-\frac{a_0}{s+b_0} \quad and \quad {\bar{\theta }(y,s)}\rightarrow 0 ~~as~~y\rightarrow \infty , \end{aligned}$$

and required solution is obtained as

25 $$\begin{aligned} \bar{\theta }(y,s)= c_{1} e^{y\sqrt{P_r \left[ \frac{k_{1}(\alpha )}{s} + k_{0}(\alpha )\right] s^{\alpha }}} + c_{2} e^{-y\sqrt{P_r \left[ \frac{k_{1}(\alpha )}{s} + k_{0}(\alpha )\right] s^{\alpha }}}, \end{aligned}$$

solution for above equation with boundary conditions given in Eq. (24) used to find unknown constants, we get

26 $$\begin{aligned} \bar{\theta }(y,s)= \left( \frac{1}{s}-\frac{a_0}{s+b_0}\right) e^{-y\sqrt{P_r \left[ \frac{k_{1}(\alpha )}{s} + k_{0}(\alpha )\right] s^{\alpha }}}, \end{aligned}$$

it can be expressed as

27 $$\begin{aligned} \bar{\theta }(y,s)&=\bar{\theta }_{1}(y,s)-a_0\bar{\theta }_{2}(y,s). \end{aligned}$$

Taking Laplace inverse which transform the above equation in time parameter, then exact solution of Eq. (27) is given by:

28 $$\begin{aligned} {\theta }(y,t)= {\theta }_{1}(y,t)-a_0{\theta }_{2}(y,t), \end{aligned}$$

where

$$\begin{aligned} {\theta }_{1}(y,t)&=\mathcal {L}^{-1}\left\{ \frac{e^{-y\sqrt{P_r \left[ \frac{k_{1}(\alpha )}{s} + k_{0}(\alpha )\right] s^{\alpha }}}}{s} \right\} . \end{aligned}$$

It is not easy to find ${\theta }_{1}(y,t)$ from exponential form, but Laplace inverse can be find if $\bar{\theta }_{1}(y,s)$ is written in series form, so for this purpose its series representations is equivalent to

$$\begin{aligned} {\theta }_{1}(y,t)&=\mathcal {L}^{-1}\left\{ \sum _{j=0}^{\infty }\frac{(-y)^{j}(P_r k_{0}(\alpha ))^{\frac{j}{2}}}{j!}.\frac{s^{\frac{j}{2}(\alpha -1)-1}}{(s+d)^{-\frac{j}{2}}} \right\} \\&=\sum _{j=0}^{\infty }\frac{(-y)^{j}(P_r k_{0}(\alpha ))^{\frac{j}{2}}}{j!}.G_{1,\frac{j}{2}(\alpha -1)-1,-\frac{j}{2}}(-d,t) , \nonumber \\ {\theta }_{2}(y,t)&=(\theta _{3} *\theta _{4})(t), \\ {\theta }_{3}(y,t)&=\mathcal {L}^{-1}\left\{ \frac{1}{s+b_0}\right\} = e^{-b_0 t},\\ {\theta }_{4}(y,t)&=\mathcal {L}^{-1}\left\{ e^{-y\sqrt{P_r \left[ \frac{k_{1}(\alpha )}{s} + k_{0}(\alpha )\right] s^{\alpha }}} \right\} ,\\&=\mathcal {L}^{-1}\left\{ \sum _{j=0}^{\infty }\frac{(-y)^{j}(P_r k_{0}(\alpha ))^{\frac{j}{2}}}{j!}.\frac{s^{\frac{j}{2}(\alpha -1)}}{(s+d)^{\frac{j}{2}}} \right\} \\&=\sum _{j=0}^{\infty }\frac{(-y)^{j}(P_r k_{0}(\alpha ))^{\frac{j}{2}}}{j!}.G_{1,\frac{j}{2}(\alpha -1),-\frac{j}{2}}(-d,t). \end{aligned}$$

### Temperature equation solution by using CF derivative operator

Employing Laplace integral transformation to Eq. (18) with conditions given in Eqs. (11, 12, 13), we have

29 $$\begin{aligned} \frac{d^{2}{\bar{ \theta }(y,s)}}{d{y}^{2}}-P_r \frac{s}{\alpha +(1-\alpha )s}{\bar{\theta }(y,s)}=0, \end{aligned}$$

with

30 $$\begin{aligned} {\bar{\theta }(0,s)}=\frac{1}{s}-\frac{a_0}{s+b_0} \quad and \quad {\bar{\theta }(y,s)}\rightarrow 0 ~~as~~y\rightarrow \infty , \end{aligned}$$

and required solution is obtained as

31 $$\begin{aligned} \bar{\theta }(y,s)= c_{1} e^{y\sqrt{\frac{P_{r} s}{(1-\alpha )s+\alpha }}} + c_{2} e^{-y\sqrt{\frac{P_{r} s}{(1-\alpha )s+\alpha }}}, \end{aligned}$$

solution for above equation with boundary conditions given in Eq. (30) used to find unknown constants, we get

32 $$\begin{aligned} \bar{\theta }(y,s)= (\frac{1}{s}-\frac{a_0}{s+b_0})e^{-y\sqrt{\frac{P_{r} s}{(1-\alpha )s+\alpha }}}, \end{aligned}$$

it can be expressed as

33 $$\begin{aligned} \bar{\theta }(y,s)&=\bar{\theta }_{1}(y,s)-a_0\bar{\theta }_{2}(y,s). \end{aligned}$$

Taking Laplace inverse which transform the above equation in time parameter, then exact solution of Eq. (33) is given by:

34 $$\begin{aligned} {\theta }(y,t)= {\theta }_{1}(y,t)-a_0{\theta }_{2}(y,t), \end{aligned}$$

where

35 $$\begin{aligned} {\theta }_{1}(y,t)&=\mathcal {L}^{-1}\left\{ \frac{e^{-y\sqrt{\frac{P_{r} s}{(1-\alpha )s+\alpha }}}}{s} \right\} \nonumber \\&=1-\frac{2P_r}{\pi }\int _{0}^{\infty }\frac{Sin(\frac{y}{\sqrt{1-\alpha }}x)}{x(P_r + x^2)} e^{-\frac{\alpha }{1-\alpha }t x^2}dx,\nonumber \\ {\theta }_{2}(y,t)&=(\theta _{3} *\theta _{4})(t), \nonumber \\ {\theta }_{3}(y,t)&=\mathcal {L}^{-1}\left\{ \frac{1}{s+b_0}\right\} = e^{-b_0 t},\nonumber \\ \bar{\theta }_{4}(y,s)&= e^{-y\sqrt{\frac{P_{r} s}{(1-\alpha )s+\alpha }}}. \end{aligned}$$

It is not easy to find ${\theta }_{4}(y,t)$ from exponential form, but Laplace inverse can be find if $\bar{\theta }_{4}(y,s)$ is written in series form, so for this purpose its series representations is equivalent to

36 $$\begin{aligned} \bar{\theta }_{4}(y,s)=\sum _{k=0}^{\infty }\sum _{j=0}^{\infty }\frac{(-1)^{j}(-y)^{k}(P_r)^{\frac{k}{2}}(\alpha )^j \Gamma (\frac{k}{2}+j)}{k! j! (1-\alpha )^{\frac{k}{2}+j}\Gamma (\frac{k}{2})}.\frac{1}{s^{j}}. \end{aligned}$$

Applying inverse transformation, we get

37 $$\begin{aligned} {\theta }_{4}(y,t)=\sum _{k=0}^{\infty }\sum _{j=0}^{\infty }\frac{(-1)^{j}(-y)^{k}(P_r)^{\frac{k}{2}}(\alpha )^j \Gamma (\frac{k}{2}+j)}{k! j! (1-\alpha )^{\frac{k}{2}+j}\Gamma (\frac{k}{2})}.\frac{t ^{j-1}}{\Gamma (j)}. \end{aligned}$$

### Temperature equation solution by using ABC derivative operator

Employing Laplace integral transformation to Eq. (21) with conditions given in Eqs. (11, 12, 13), we have

38 $$\begin{aligned} \frac{d^{2}{\bar{ \theta }(y,s)}}{d{y}^{2}}-P_r \frac{s^{\alpha }}{\alpha +(1-\alpha )s^{\alpha }}{\bar{\theta }(y,s)}=0, \end{aligned}$$

with

39 $$\begin{aligned} {\bar{\theta }(0,s)}=\frac{1}{s}-\frac{a_0}{s+b_0} \quad and \quad {\bar{\theta }(y,s)}\rightarrow 0 ~~as~~y\rightarrow \infty , \end{aligned}$$

and required solution is obtained as

40 $$\begin{aligned} \bar{\theta }(y,s)= c_{1} e^{y\sqrt{\frac{P_{r} s^{\alpha }}{(1-\alpha )s^{\alpha }+\alpha }}} + c_{2} e^{-y\sqrt{\frac{P_{r} s^{\alpha }}{(1-\alpha )s^{\alpha }+\alpha }}}, \end{aligned}$$

solution for above equation with boundary conditions given in Eq. (30) used to find unknown constants, we get

41 $$\begin{aligned} \bar{\theta }(y,s)= (\frac{1}{s}-\frac{a_0}{s+b_0})e^{-y\sqrt{\frac{P_{r} s^{\alpha }}{(1-\alpha )s^{\alpha }+\alpha }}}, \end{aligned}$$

it can be expressed as

42 $$\begin{aligned} \bar{\theta }(y,s)&=\bar{\theta }_{5}(y,s)-a_0\bar{\theta }_{6}(y,s). \end{aligned}$$

Taking Laplace inverse which transform the above equation in time parameter, then exact solution of Eq. (42) is given by:

43 $$\begin{aligned} {\theta }(y,t)= {\theta }_{5}(y,t)-a_0{\theta }_{6}(y,t), \end{aligned}$$

where

44 $$\begin{aligned} {\theta }_{5}(y,t)&=\mathcal {L}^{-1}\left\{ \frac{e^{-y\sqrt{\frac{P_{r} s^{\alpha }}{(1-\alpha )s^{\alpha }+\alpha }}}}{s} \right\} . \end{aligned}$$

It is not easy to find ${\theta }_{5}(y,t)$ from exponential form, but Laplace inverse can be find if $\bar{\theta }_{5}(y,s)$ is written in series form, so for this purpose its series representations is equivalent to

45 $$\begin{aligned} \bar{\theta }_{4}(y,s)=\sum _{k=0}^{\infty }\sum _{j=0}^{\infty }\frac{(-1)^{j}(-y)^{k}(P_r)^{\frac{k}{2}}(\alpha )^j \Gamma (\frac{k}{2}+j)}{k! j! (1-\alpha )^{\frac{k}{2}+j}\Gamma (\frac{k}{2})}.\frac{1}{s^{j\alpha +1}}. \end{aligned}$$

Applying inverse transformation, we get

46 $$\begin{aligned} {\theta }_{4}(y,t)&=\sum _{k=0}^{\infty }\sum _{j=0}^{\infty }\frac{(-1)^{j}(-y)^{k}(P_r)^{\frac{k}{2}}(\alpha )^j \Gamma (\frac{k}{2}+j)}{k! j! (1-\alpha )^{\frac{k}{2}+j}\Gamma (\frac{k}{2})}.\frac{t ^{j\alpha }}{\Gamma (j\alpha +1)}. \end{aligned}$$

47 $$\begin{aligned} {\theta }_{6}(y,t)&=(\theta _{7} *\theta _{8})(t), \nonumber \\ {\theta }_{7}(y,t)&=\mathcal {L}^{-1}\left\{ \frac{1}{s+b_0}\right\} = e^{-b_0 t},\nonumber \\ \bar{\theta }_{8}(y,s)&= e^{-y\sqrt{\frac{P_{r} s^{\alpha }}{(1-\alpha )s^{\alpha }+\alpha }}}, \end{aligned}$$

It is not easy to find ${\theta }_{8}(y,t)$ from exponential form, but Laplace inverse can be find if $\bar{\theta }_{8}(y,s)$ is written in series form, so for this purpose its series representations is equivalent to

48 $$\begin{aligned} \bar{\theta }_{4}(y,s)=\sum _{k=0}^{\infty }\sum _{j=0}^{\infty }\frac{(-1)^{j}(-y)^{k}(P_r)^{\frac{k}{2}}(\alpha )^j \Gamma (\frac{k}{2}+j)}{k! j! (1-\alpha )^{\frac{k}{2}+j}\Gamma (\frac{k}{2})}.\frac{1}{s^{j\alpha }}. \end{aligned}$$

Applying inverse transformation, we get

49 $$\begin{aligned} {\theta }_{4}(y,t)=\sum _{k=0}^{\infty }\sum _{j=0}^{\infty }\frac{(-1)^{j}(-y)^{k}(P_r)^{\frac{k}{2}}(\alpha )^j \Gamma (\frac{k}{2}+j)}{k! j! (1-\alpha )^{\frac{k}{2}+j}\Gamma (\frac{k}{2})}.\frac{t ^{j\alpha -1}}{\Gamma (j\alpha )}. \end{aligned}$$

### Concentration equation solution by using CPC derivative operator

Solving Eq. (16) using (11), (12) and (13), then it becomes

50 $$\begin{aligned} \frac{d^{2}{\bar{C}(y,s)}}{d{y}^{2}}-\left( {S_c}\left[ \frac{k_{1}(\alpha )}{s} + k_{0}(\alpha )\right] s^{\alpha }+\lambda \right) {\bar{C}(y,s)}=0, \end{aligned}$$

with conditions transformed as

51 $$\begin{aligned} {\bar{C}(y,s)}\rightarrow 0 ~~as~~y\rightarrow \infty \quad and \quad {\bar{C}(0,s)}=\frac{1}{s}, \end{aligned}$$

the solution for concentration is described as:

52 $$\begin{aligned} \bar{C}(y,s)= c_{3} e^{{y}\sqrt{{S_c}\left[ \frac{k_{1}(\alpha )}{s} + k_{0}(\alpha )\right] s^{\alpha }+\lambda }} + c_{4} e^{-{y}\sqrt{{S_c}\left[ \frac{k_{1}(\alpha )}{s} + k_{0}(\alpha )\right] s^{\alpha }+\lambda }}. \end{aligned}$$

Applying conditions given in Eq. (57) for concentration then the above solution has the form

53 $$\begin{aligned} \bar{C}(y,s)= \frac{1}{s}e^{-{y}\sqrt{{S_c}\left[ \frac{k_{1}(\alpha )}{s} + k_{0}(\alpha )\right] s^{\alpha }+\lambda }}. \end{aligned}$$

It is not easy to written the concentration solution *C*(*y*, *t*) from exponential form, but Laplace inverse can be find if $\bar{C}(y,s)$ is written in series form, so for this purpose its series representations is equivalent to

54 $$\begin{aligned} \bar{C}(y,s)=\sum _{l=0}^{\infty }\sum _{m=0}^{\infty }\frac{(-y)^l(\lambda )^{\frac{l}{2}-m}(S_c k_{0}(\alpha ))^m \Gamma (\frac{l}{2}+1)}{l!m! \Gamma (\frac{l}{2}-m+1)}.\frac{s^{m(\alpha -1)-1}}{(s+d)^{-m}}. \end{aligned}$$

For concentration solution, taking Laplace inverse of Eq. (60), so finally it takes the form

55 $$\begin{aligned} C(y,t)=\sum _{l=0}^{\infty }\sum _{m=0}^{\infty }\frac{(-y)^l(\lambda )^{\frac{l}{2}-m}(S_c k_{0}(\alpha ))^m \Gamma (\frac{l}{2}+1)}{l!m! \Gamma (\frac{l}{2}-m+1)}.G_{1,m(\alpha -1)-1,-m}(-d,t). \end{aligned}$$

### Concentration equation solution by using CF derivative operator

Solving Eq. (19) using (11), (12) and (13), then it becomes

56 $$\begin{aligned} \frac{d^{2}{\bar{C}(y,s)}}{d{y}^{2}}-\left( {S_c}\frac{ s}{(1-\alpha )s+\alpha }+\lambda \right) {\bar{C}(y,s)}=0, \end{aligned}$$

with conditions transformed as

57 $$\begin{aligned} {\bar{C}(y,s)}\rightarrow 0 ~~as~~y\rightarrow \infty \quad and \quad {\bar{C}(0,s)}=\frac{1}{s}, \end{aligned}$$

the solution for concentration is described as:

58 $$\begin{aligned} \bar{C}(y,s)= c_{3} e^{{y}\sqrt{{S_c}\frac{ s}{(1-\alpha )s+\alpha }+\lambda }} + c_{4} e^{-{y}\sqrt{{S_c}\frac{ s}{(1-\alpha )s+\alpha }+\lambda }}. \end{aligned}$$

Applying conditions given in Eq. (57) for concentration then the above solution has the form

59 $$\begin{aligned} \bar{C}(y,s)= \frac{1}{s}e^{-{y}\sqrt{{S_c}\frac{ s}{(1-\alpha )s+\alpha }+\lambda }}. \end{aligned}$$

It is not easy to written the concentration solution *C*(*y*, *t*) from exponential form, but Laplace inverse can be find if $\bar{C}(y,s)$ is written in series form, so for this purpose its series representations is equivalent to

60 $$\begin{aligned} \bar{C}(y,s)=\sum _{l=0}^{\infty }\sum _{m=0}^{\infty }\sum _{n=0}^{\infty }\frac{(-y)^l(\lambda )^{\frac{l}{2}-m}(S_c)^m \alpha ^n \Gamma (\frac{l}{2}+1)\Gamma (m+n)}{l!m!n! (1-\alpha )^{m+n} \Gamma (\frac{l}{2}-m+1)\Gamma (m)}.\frac{1}{s^{1+n}}. \end{aligned}$$

For concentration solution, taking Laplace inverse of Eq. (60), so finally it takes the form

61 $$\begin{aligned} C(y,t)=\sum _{l=0}^{\infty }\sum _{m=0}^{\infty }\sum _{n=0}^{\infty }\frac{(-y)^l(\lambda )^{\frac{l}{2}-m}(S_c)^m \alpha ^n \Gamma (\frac{l}{2}+1)\Gamma (m+n)}{l!m!n! (1-\alpha )^{m+n} \Gamma (\frac{l}{2}-m+1)\Gamma (m)}.\frac{t^{n}}{\Gamma (1+n)}. \end{aligned}$$

### Concentration equation solution by using ABC derivative operator

Solving Eq. (22) using (11), (12) and (13), then it becomes

62 $$\begin{aligned} \frac{d^{2}{\bar{C}(y,s)}}{d{y}^{2}}-\left( {S_c}\frac{ s^{\alpha }}{(1-\alpha )s^{\alpha }+\alpha }+\lambda \right) {\bar{C}(y,s)}=0, \end{aligned}$$

with conditions transformed as

63 $$\begin{aligned} {\bar{C}(y,s)}\rightarrow 0 ~~as~~y\rightarrow \infty \quad and \quad {\bar{C}(0,s)}=\frac{1}{s}, \end{aligned}$$

the solution for concentration is described as:

64 $$\begin{aligned} \bar{C}(y,s)= c_{3} e^{{y}\sqrt{{S_c}\frac{ s^{\alpha }}{(1-\alpha )s^{\alpha }+\alpha }+\lambda }} + c_{4} e^{-{y}\sqrt{{S_c}\frac{s^{\alpha }}{(1-\alpha )s^{\alpha }+\alpha }+\lambda }}. \end{aligned}$$

Applying conditions given in Eq. (57) for concentration then the above solution has the form

65 $$\begin{aligned} \bar{C}(y,s)= \frac{1}{s}e^{-{y}\sqrt{{S_c}\frac{ s^{\alpha }}{(1-\alpha )s^{\alpha }+\alpha }+\lambda }}. \end{aligned}$$

It is not easy to written the concentration solution *C*(*y*, *t*) from exponential form, but Laplace inverse can be find if $\bar{C}(y,s)$ is written in series form, so for this purpose its series representations is equivalent to

66 $$\begin{aligned} \bar{C}(y,s)=\sum _{l=0}^{\infty }\sum _{m=0}^{\infty }\sum _{n=0}^{\infty }\frac{(-y)^l(\lambda )^{\frac{l}{2}-m}(S_c)^m \alpha ^n \Gamma (\frac{l}{2}+1)\Gamma (m+n)}{l!m!n! (1-\alpha )^{m+n} \Gamma (\frac{l}{2}-m+1)\Gamma (m)}.\frac{1}{s^{n{\alpha }+1}}. \end{aligned}$$

For concentration solution, taking Laplace inverse of Eq. (66), so finally it takes the form

67 $$\begin{aligned} C(y,t)=\sum _{l=0}^{\infty }\sum _{m=0}^{\infty }\sum _{n=0}^{\infty }\frac{(-y)^l(\lambda )^{\frac{l}{2}-m}(S_c)^m \alpha ^n \Gamma (\frac{l}{2}+1)\Gamma (m+n)}{l!m!n! (1-\alpha )^{m+n} \Gamma (\frac{l}{2}-m+1)\Gamma (m)}.\frac{t^{n{\alpha }}}{\Gamma (n{\alpha }+1)}. \end{aligned}$$

### Exact solution of fluid velocity by using CF derivative operator

Solution of Eq. (17) with the application of Laplace integral transformation, then its solution in the following form

68 $$\begin{aligned} \left( a+(1+b)\frac{ s}{(1-\alpha )s+\alpha }\right) \bar{u}(y,s)=\left( 1+\alpha _2\frac{ s}{(1-\alpha )s+\alpha }\right) \frac{d^{2}{\bar{u}(y,s)}}{d{y}^{2}} +G_{r}\bar{\theta }(y,s)+G_{m} \bar{C}(y,s), \end{aligned}$$

rearrange the above equation then it can be written as:

69 $$\begin{aligned} \frac{d^{2}{\bar{u}(y,s)}}{d{y}^{2}}-\left( \frac{b_1 s+a_1}{b_2 s+ \alpha }\right) \bar{u}(y,s)= - \left( \frac{b_3 s+\alpha }{b_2 s+ \alpha }\right) \left( G_{r}\bar{\theta }(y,s)+G_{m}{\bar{C}(y,s)}\right) , \end{aligned}$$

by using the Eqs. (32) and (59) in Eq. (69) then the solution in general form is represented as:

70 $$\begin{aligned} \bar{u}(y,s)&=c_{5}e^{y\sqrt{\frac{b_1 s+a_1}{b_2 s+ \alpha }}}+c_{6}e^{-y\sqrt{\frac{b_1 s+a_1}{b_2 s+ \alpha }}}-\left( \frac{G_{r}\left( b_3 s + \alpha \right) ^2\left( \frac{1}{s}-\frac{a_0}{s+b_0}\right) e^{-y\sqrt{\frac{P_r s}{b_3 s +\alpha }}}}{s\left( b_2 s+ \alpha \right) P_r-(b_1 s+ a_1)(b_3 s+ \alpha ) }\right) \nonumber \\&-\left( \frac{G_{m}\left( b_3 s + \alpha \right) ^2\left( \frac{1}{s}\right) e^{-y\sqrt{\frac{S_c s}{b_3 s +\alpha }}}}{s\left( b_2 s+ \alpha \right) S_c-(b_1 s+ a_1)(b_3 s+ \alpha ) }\right) , \end{aligned}$$

to determine unknowns $c_{5}$ and $c_{6}$, using ${\bar{u}(0,s)}=\frac{1}{s-i \epsilon }$ and ${\bar{u}(y,s)}\rightarrow 0$ as $y\rightarrow \infty$, we get

71 $$\begin{aligned} \bar{u}(y,s)&=\frac{1}{s-i \epsilon }e^{-y\sqrt{\frac{b_1 s+a_1}{b_2 s+ \alpha }}}+\left( \frac{G_{r}\left( b_3 s + \alpha \right) ^2\left( \frac{1}{s}-\frac{a_0}{s+b_0}\right) }{s\left( b_2 s+ \alpha \right) P_r-(b_1 s+ a_1)(b_3 s+ \alpha ) }\right) \left[ e^{-y\sqrt{\frac{b_1 s+a_1}{b_2 s+ \alpha }}}-e^{-y\sqrt{\frac{P_r s}{b_3 s +\alpha }}} \right] \nonumber \\&+\left( \frac{G_{m}\left( b_3 s + \alpha \right) ^2\left( \frac{1}{s}\right) }{s\left( b_2 s+ \alpha \right) S_c-(b_1 s+ a_1)(b_3 s+ \alpha ) }\right) \left[ e^{-y\sqrt{\frac{b_1 s+a_1}{b_2 s+ \alpha }}}-e^{-y\sqrt{\frac{S_c s}{b_3 s +\alpha }}} \right] , \end{aligned}$$

the above expression can be expressed in the following way

72 $$\begin{aligned} \bar{u}\left( y,s\right)&=\bar{E}\left( y,s\right) \bar{F}\left( y,s\right) +G_r\bar{P}\left( y,s\right) \left[ \bar{D}\left( y,s\right) -a_0\bar{L}\left( y,s\right) -\bar{\theta }\left( y,s\right) \right] \nonumber \\&+G_m\bar{R}\left( y,s\right) \left[ \bar{D}\left( y,s\right) -\bar{C}\left( y,s\right) \right] . \end{aligned}$$

Applying Laplace inverse to write the solution for momentum equation as:

73 $$\begin{aligned} {u}\left( y,t\right)&=\left( E *F\right) (t)+G_r\left[ \left( P *D\right) (t) -a_0 \left( P *L\right) (t) - \left( P *\theta \right) (t) \right] \nonumber \\&+G_m\left[ \left( R *D\right) (t)-\left( R *C\right) (t) \right] , \end{aligned}$$

where

74 $$\begin{aligned} \bar{F}\left( y,s\right)&= e^{-y\sqrt{\frac{b_1 s+a_1}{b_2 s+ \alpha }}}, \end{aligned}$$

first express the $\bar{F}\left( y,s\right)$ in series form ,

75 $$\begin{aligned} \bar{F}(y,s)=\sum _{v=1}^{\infty }\sum _{p=0}^{\infty }\sum _{h=0}^{\infty }\frac{(-1)^h (-y)^v (a_2)^{p} (b_4)^{\frac{v}{2}-p}(\alpha )^h \Gamma (\frac{v}{2}+1)\Gamma (p+h)}{v!\quad p!\quad h!\quad (b_2)^{p+h} \quad \Gamma (\frac{v}{2}-p+1)\Gamma (h) }.\frac{1}{s^{p+h}}. \end{aligned}$$

Employing Laplace inverse then it takes the form as:

76 $$\begin{aligned} {F}(y,t)=\sum _{v=1}^{\infty }\sum _{p=0}^{\infty }\sum _{h=0}^{\infty }\frac{(-1)^h (-y)^v (a_2)^{p} (b_4)^{\frac{v}{2}-p}(\alpha )^h \Gamma (\frac{v}{2}+1)\Gamma (p+h)}{v!\quad p!\quad h!\quad (b_2)^{p+h} \quad \Gamma (\frac{v}{2}-p+1)\Gamma (h) }.\frac{t^{p+h-1}}{\Gamma (p+h)}. \end{aligned}$$

Similarly, *D*(*y*, *t*) is calculated as:

77 $$\begin{aligned} \bar{D}\left( y,s\right)&=\frac{1}{s}\bar{F}\left( y,s\right) ,\nonumber \\&=\frac{1}{s} \sum _{v=1}^{\infty }\sum _{p=0}^{\infty }\sum _{h=0}^{\infty }\frac{(-1)^h (-y)^v (a_2)^{p} (b_4)^{\frac{v}{2}-p}(\alpha )^h \Gamma (\frac{v}{2}+1)\Gamma (p+h)}{v!\quad p!\quad h!\quad (b_2)^{p+h} \quad \Gamma (\frac{v}{2}-p+1)\Gamma (h) }.\frac{1}{s^{p+h}}, \nonumber \\&= \sum _{v=1}^{\infty }\sum _{p=0}^{\infty }\sum _{h=0}^{\infty }\frac{(-1)^h (-y)^v (a_2)^{p} (b_4)^{\frac{v}{2}-p}(\alpha )^h \Gamma (\frac{v}{2}+1)\Gamma (p+h)}{v!\quad p!\quad h!\quad (b_2)^{p+h} \quad \Gamma (\frac{v}{2}-p+1)\Gamma (h) }.\frac{1}{s^{p+h+1}}, \nonumber \\ {D}(y,t)&=\sum _{v=1}^{\infty }\sum _{p=0}^{\infty }\sum _{h=0}^{\infty }\frac{(-1)^h (-y)^v (a_2)^{p} (b_4)^{\frac{v}{2}-p}(\alpha )^h \Gamma (\frac{v}{2}+1)\Gamma (p+h)}{v!\quad p!\quad h!\quad (b_2)^{p+h} \quad \Gamma (\frac{v}{2}-p+1)\Gamma (h) }.\frac{t^{p+h}}{\Gamma (p+h+1)}, \end{aligned}$$

and

78 $$\begin{aligned} {E}\left( y,t\right)&= \mathcal {L}^{-1}\left\{ \frac{1}{s-i \epsilon }\right\} = e^{i \epsilon t},\nonumber \\ {J}\left( y,t\right)&= \mathcal {L}^{-1}\left\{ \frac{1}{s+ b_0}\right\} = e^{-b_0 t},\nonumber \\ \bar{L}\left( y,s\right)&= \bar{J}\left( y,s\right) \bar{F}\left( y,s\right) ,\nonumber \\ {L}\left( y,t\right)&= (J *F)(t)=\int _{0}^{t}F(t)J(t - t)dt. \end{aligned}$$

Next consider $\bar{P}(y,s)$ to calculate *P*(*y*, *t*), we have

79 $$\begin{aligned} \bar{P}\left( y,s\right)&=(b_3 s + \alpha )^2\frac{1}{(Pr b_2 -b_1 b_3) s^{2}- (a_1 b_3+ b_1 \alpha - P_r \alpha ) s - a_1\alpha }, \nonumber \\&=(b_3 s + \alpha )^2\frac{1}{\beta s^{2}- \gamma s - \delta }, \nonumber \\&=b_3^2 \sum _{n=0}^{\infty }\frac{(\delta )^{n}}{(\beta )^{n+1}}\frac{(s)^{-n+1}}{(s - \frac{\gamma }{\beta })^{n+1}} + 2 b_3 \alpha \sum _{n=0}^{\infty }\frac{(\delta )^{n}}{(\beta )^{n+1}}\frac{(s)^{-n}}{(s - \frac{\gamma }{\beta })^{n+1}} +\alpha ^2 \sum _{n=0}^{\infty }\frac{(\delta )^{n}}{(\beta )^{n+1}}\frac{(s)^{-n-1}}{(s - \frac{\gamma }{\beta })^{n+1}},\nonumber \\ {P}\left( y,t\right)&=\sum _{n=0}^{\infty }\frac{(\delta )^{n}}{(\beta )^{n+1}}\left[ b_3^2G_{1,-n+1, n + 1}(\frac{\gamma }{\beta },t) +2 b_3 \alpha G_{1,-n, n + 1}(\frac{\gamma }{\beta },t) +\alpha ^2G_{1,-n-1, n + 1}(\frac{\gamma }{\beta },t)\right] . \end{aligned}$$

Similarly, we can calculate *R*(*y*, *t*), so consider

80 $$\begin{aligned} \bar{R}\left( y,s\right)&=(b_3 s + \alpha )^2\frac{1}{(S_c b_2 -b_1 b_3) s^{2}- (a_1 b_3+ b_1 \alpha - S_c \alpha ) s - a_1\alpha }, \nonumber \\&=(b_3 s + \alpha )^2\frac{1}{\chi s^{2}- \xi s - \delta }, \nonumber \\&=b_3^2 \sum _{n=0}^{\infty }\frac{(\delta )^{n}}{(\chi )^{n+1}}\frac{(s)^{-n+1}}{(s - \frac{\xi }{\chi })^{n+1}} + 2 b_3 \alpha \sum _{n=0}^{\infty }\frac{(\delta )^{n}}{(\chi )^{n+1}}\frac{(s)^{-n}}{(s - \frac{\xi }{\chi })^{n+1}} +\alpha ^2 \sum _{n=0}^{\infty }\frac{(\delta )^{n}}{(\chi )^{n+1}}\frac{(s)^{-n-1}}{(s - \frac{\xi }{\chi })^{n+1}},\nonumber \\ {R}\left( y,t\right)&=\sum _{n=0}^{\infty }\frac{(\delta )^{n}}{(\beta )^{n+1}}\left[ b_3^2G_{1,-n+1, n + 1}(\frac{\xi }{\chi },t) +2 b_3 \alpha G_{1,-n, n + 1}(\frac{\xi }{\chi },t) +\alpha ^2G_{1,-n-1, n + 1}(\frac{\xi }{\chi },t)\right] , \end{aligned}$$

### Exact solution of fluid velocity by using ABC derivative operator

Solution of Eq. (20) with the application of Laplace integral transformation, then its solution in the following form

81 $$\begin{aligned} \left( a+(1+b)\frac{ s^{\alpha }}{(1-\alpha )s^{\alpha }+\alpha }\right) \bar{u}(y,s)=\left( 1+\alpha _2\frac{s^{\alpha }}{(1-\alpha )s^{\alpha }+\alpha }\right) \frac{d^{2}{\bar{u}(y,s)}}{d{y}^{2}} +G_{r}\bar{\theta }(y,s)+G_{m} \bar{C}(y,s), \end{aligned}$$

rearrange the above equation then it can be written as:

82 $$\begin{aligned} \frac{d^{2}{\bar{u}(y,s)}}{d{y}^{2}}-\left( \frac{b_1s^{\alpha }+a_1}{b_2 s^{\alpha }+ \alpha }\right) \bar{u}(y,s)= - \left( \frac{b_3s^{\alpha }+\alpha }{b_2 s^{\alpha }+ \alpha }\right) \left( G_{r}\bar{\theta }(y,s)+G_{m}{\bar{C}(y,s)}\right) , \end{aligned}$$

by using the Eqs. (41) and (65) in Eq. (82) then the solution in general form is represented as:

83 $$\begin{aligned} \bar{u}(y,s)&=c_{11}e^{y\sqrt{\frac{b_1 s^{\alpha }+a_1}{b_2 s^{\alpha }+ \alpha }}}+c_{12}e^{-y\sqrt{\frac{b_1 s^{\alpha }+a_1}{b_2 s^{\alpha }+ \alpha }}}-\left( \frac{G_{r}\left( b_3 s^{\alpha } + \alpha \right) ^2\left( \frac{1}{s}-\frac{a_0}{s+b_0}\right) e^{-y\sqrt{\frac{P_r s^{\alpha }}{b_3 s^{\alpha } +\alpha }}}}{s^{\alpha }\left( b_2 s^{\alpha }+ \alpha \right) P_r-(b_1 s^{\alpha }+ a_1)(b_3 s^{\alpha }+ \alpha ) }\right) \nonumber \\&-\left( \frac{G_{m}\left( b_3 s^{\alpha } + \alpha \right) ^2\left( \frac{1}{s}\right) e^{-y\sqrt{\frac{S_c s^{\alpha }}{b_3 s^{\alpha } +\alpha }}}}{s^{\alpha }\left( b_2 s^{\alpha }+ \alpha \right) S_c-(b_1 s^{\alpha }+ a_1)(b_3 s^{\alpha }+ \alpha ) }\right) , \end{aligned}$$

to determine unknowns $c_{11}$ and $c_{12}$, using ${\bar{u}(0,s)}=\frac{1}{s-i \epsilon }$ and ${\bar{u}(y,s)}\rightarrow 0$ as $y\rightarrow \infty$, we get

84 $$\begin{aligned} \bar{u}(y,s)&=\frac{1}{s-i \epsilon }e^{-y\sqrt{\frac{b_1 s^{\alpha }+a_1}{b_2 s^{\alpha }+ \alpha }}}+\left( \frac{G_{r}\left( b_3 s^{\alpha } + \alpha \right) ^2\left( \frac{1}{s}-\frac{a_0}{s+b_0}\right) }{s^{\alpha }\left( b_2 s^{\alpha }+ \alpha \right) P_r-(b_1 s^{\alpha }+ a_1)(b_3 s^{\alpha }+ \alpha ) }\right) \left[ e^{-y\sqrt{\frac{b_1 s^{\alpha }+a_1}{b_2 s^{\alpha }+ \alpha }}}-e^{-y\sqrt{\frac{P_r s^{\alpha }}{b_3 s^{\alpha } +\alpha }}} \right] \nonumber \\&+\left( \frac{G_{m}\left( b_3 s^{\alpha } + \alpha \right) ^2\left( \frac{1}{s}\right) }{s^{\alpha }\left( b_2 s^{\alpha }+ \alpha \right) S_c-(b_1 s^{\alpha }+ a_1)(b_3 s^{\alpha }+ \alpha ) }\right) \left[ e^{-y\sqrt{\frac{b_1 s^{\alpha }+a_1}{b_2 s^{\alpha }+ \alpha }}}-e^{-y\sqrt{\frac{S_c s^{\alpha }}{b_3 s^{\alpha } +\alpha }}} \right] , \end{aligned}$$

the above expression can be expressed in the following way

85 $$\begin{aligned} \bar{u}\left( y,s\right)&=\bar{E_1}\left( y,s\right) \bar{F_1}\left( y,s\right) +G_r\bar{P_1}\left( y,s\right) \left[ \bar{D_1}\left( y,s\right) -a_0\bar{L_1}\left( y,s\right) -\bar{\theta }\left( y,s\right) \right] \nonumber \\&+G_m\bar{R_1}\left( y,s\right) \left[ \bar{D_1}\left( y,s\right) -\bar{C}\left( y,s\right) \right] . \end{aligned}$$

Applying Laplace inverse to write the solution for momentum equation as:

86 $$\begin{aligned} {u}\left( y,t\right)&=\left( E_1 *F_1 \right) (t)+G_r\left[ \left( P_1 *D_1 \right) (t) -a_0 \left( P_1 *L_1 \right) (t) - \left( P_1 *\theta \right) (t) \right] \nonumber \\&+G_m\left[ \left( R_1 *D_1 \right) (t)-\left( R_1 *C\right) (t) \right] , \end{aligned}$$

where

87 $$\begin{aligned} \bar{F_1}\left( y,s\right)&= e^{-y\sqrt{\frac{b_1 s^{\alpha }+a_1}{b_2 s^{\alpha }+ \alpha }}}, \end{aligned}$$

first express the $\bar{F_1}\left( y,s\right)$ in series form,

88 $$\begin{aligned} \bar{F_1}(y,s)=\sum _{v=1}^{\infty }\sum _{p=0}^{\infty }\sum _{h=0}^{\infty }\frac{(-1)^h (-y)^v (a_2)^{p} (b_4)^{\frac{v}{2}-p}(\alpha )^h \Gamma (\frac{v}{2}+1)\Gamma (p+h)}{v!\quad p!\quad h!\quad (b_2)^{p+h} \quad \Gamma (\frac{v}{2}-p+1)\Gamma (h) }.\frac{1}{s^{{\alpha } p+{\alpha } h}}. \end{aligned}$$

Employing Laplace inverse then it takes the form as:

89 $$\begin{aligned} {F}(y,t)=\sum _{v=1}^{\infty }\sum _{p=0}^{\infty }\sum _{h=0}^{\infty }\frac{(-1)^h (-y)^v (a_2)^{p} (b_4)^{\frac{v}{2}-p}(\alpha )^h \Gamma (\frac{v}{2}+1)\Gamma (p+h)}{v!\quad p!\quad h!\quad (b_2)^{p+h} \quad \Gamma (\frac{v}{2}-p+1)\Gamma (h) }.\frac{t^{{\alpha } p+{\alpha } h-1}}{\Gamma ({\alpha } p+{\alpha } h)}. \end{aligned}$$

Similarly, ${D_1}(y,t)$ is calculated as:

90 $$\begin{aligned} \bar{D_1}\left( y,s\right)&=\frac{1}{s}\bar{F_1}\left( y,s\right) ,\nonumber \\&=\frac{1}{s} \sum _{v=1}^{\infty }\sum _{p=0}^{\infty }\sum _{h=0}^{\infty }\frac{(-1)^h (-y)^v (a_2)^{p} (b_4)^{\frac{v}{2}-p}(\alpha )^h \Gamma (\frac{v}{2}+1)\Gamma (p+h)}{v!\quad p!\quad h!\quad (b_2)^{p+h} \quad \Gamma (\frac{v}{2}-p+1)\Gamma (h) }.\frac{1}{s^{{\alpha } p+{\alpha } h}}, \nonumber \\&= \sum _{v=1}^{\infty }\sum _{p=0}^{\infty }\sum _{h=0}^{\infty }\frac{(-1)^h (-y)^v (a_2)^{p} (b_4)^{\frac{v}{2}-p}(\alpha )^h \Gamma (\frac{v}{2}+1)\Gamma (p+h)}{v!\quad p!\quad h!\quad (b_2)^{p+h} \quad \Gamma (\frac{v}{2}-p+1)\Gamma (h) }.\frac{1}{s^{{\alpha } p+{\alpha } h+1}}, \nonumber \\ {D_1}(y,t)&=\sum _{v=1}^{\infty }\sum _{p=0}^{\infty }\sum _{h=0}^{\infty }\frac{(-1)^h (-y)^v (a_2)^{p} (b_4)^{\frac{v}{2}-p}(\alpha )^h \Gamma (\frac{v}{2}+1)\Gamma (p+h)}{v!\quad p!\quad h!\quad (b_2)^{p+h} \quad \Gamma (\frac{v}{2}-p+1)\Gamma (h) }.\frac{t^{{\alpha } p+{\alpha } h}}{\Gamma ({\alpha } p+{\alpha } h+1)}, \end{aligned}$$

and

91 $$\begin{aligned} {E_1}\left( y,t\right)&= \mathcal {L}^{-1}\left\{ \frac{1}{s-i \epsilon }\right\} = e^{i \epsilon t},\nonumber \\ {J_1}\left( y,t\right)&= \mathcal {L}^{-1}\left\{ \frac{1}{s+ b_0}\right\} = e^{-b_0 t},\nonumber \\ \bar{L_1}\left( y,s\right)&= \bar{J_1}\left( y,s\right) \bar{F}\left( y,s\right) ,\nonumber \\ {L_1}\left( y,t\right)&= (J_1 *F_1)(t)=\int _{0}^{t}F_1(t)J_1(t - t)dt. \end{aligned}$$

Next consider $\bar{P_1}(y,s)$ to calculate ${P_1}(y,t)$, we have

92 $$\begin{aligned} \bar{P_1}\left( y,s\right)&=(b_3 s^{\alpha } + \alpha )^2\frac{1}{(Pr b_2 -b_1 b_3) s^{2{\alpha }}- (a_1 b_3+ b_1 \alpha - P_r \alpha ) s^{\alpha } - a_1\alpha }, \nonumber \\&=(b_3 s^{\alpha } + \alpha )^2\frac{1}{\beta s^{2 {\alpha }}- \gamma s^{\alpha } - \delta }, \nonumber \\&=b_3^2 \sum _{n=0}^{\infty }\frac{(\delta )^{n}}{(\beta )^{n+1}}\frac{(s)^{-n{\alpha }+{\alpha }}}{(s^{\alpha } - \frac{\gamma }{\beta })^{n+1}} + 2 b_3 \alpha \sum _{n=0}^{\infty }\frac{(\delta )^{n}}{(\beta )^{n+1}}\frac{(s)^{-n{\alpha }}}{(s^{\alpha } - \frac{\gamma }{\beta })^{n+1}} +\alpha ^2 \sum _{n=0}^{\infty }\frac{(\delta )^{n}}{(\beta )^{n+1}}\frac{(s)^{-n{\alpha }-{\alpha }}}{(s^{\alpha } - \frac{\gamma }{\beta })^{n+1}},\nonumber \\ {P_1}\left( y,t\right)&=\sum _{n=0}^{\infty }\frac{(\delta )^{n}}{(\beta )^{n+1}}\left[ b_3^2G_{1,-n{\alpha }+{\alpha }, n + 1}(\frac{\gamma }{\beta },t) +2 b_3 \alpha G_{1,-n{\alpha }, n + 1}(\frac{\gamma }{\beta },t) +\alpha ^2G_{1,-n{\alpha }-{\alpha }, n + 1}(\frac{\gamma }{\beta },t)\right] . \end{aligned}$$

Similarly, we can calculate *R*(*y*, *t*), so consider

93 $$\begin{aligned} \bar{R_1}\left( y,s\right)&=(b_3 s^{\alpha } + \alpha )^2\frac{1}{(S_c b_2 -b_1 b_3) s^{2{\alpha }}- (a_1 b_3+ b_1 \alpha - S_c \alpha ) s^{\alpha } - a_1\alpha }, \nonumber \\&=(b_3 s^{\alpha } + \alpha )^2\frac{1}{\chi s^{2{\alpha }}- \xi s^{\alpha } - \delta }, \nonumber \\&=b_3^2 \sum _{n=0}^{\infty }\frac{(\delta )^{n}}{(\chi 
)^{n+1}}\frac{(s)^{-n{\alpha }+{\alpha }}}{(s^{\alpha } - \frac{\xi }{\chi })^{n+1}} + 2 b_3 \alpha \sum _{n=0}^{\infty }\frac{(\delta )^{n}}{(\chi )^{n+1}}\frac{(s)^{-n{\alpha }}}{(s^{\alpha } - \frac{\xi }{\chi })^{n+1}} +\alpha ^2 \sum _{n=0}^{\infty }\frac{(\delta )^{n}}{(\chi )^{n+1}}\frac{(s)^{-n{\alpha }-{\alpha }}}{(s^{\alpha } - \frac{\xi }{\chi })^{n+1}},\nonumber \\ {R_1}\left( y,t\right)&=\sum _{n=0}^{\infty }\frac{(\delta )^{n}}{(\chi )^{n+1}}\left[ b_3^2G_{1,-n{\alpha }+{\alpha }, n + 1}(\frac{\xi }{\chi },t) +2 b_3 \alpha G_{1,-n{\alpha }, n + 1}(\frac{\xi }{\chi },t) +\alpha ^2G_{1,-n{\alpha }-{\alpha }, n + 1}(\frac{\xi }{\chi },t)\right] , \end{aligned}$$

where

94 $$\begin{aligned} \beta&= P_rb_2 - b_1 b_3, \quad \gamma =a_1 b_3 +b_1 \alpha - P_r \alpha ,\quad \delta = a_1 \alpha ,\nonumber \\ \chi&= S_c b_2 - b_1 b_3, \quad \xi =a_1 b_3 +b_1 \alpha - S_c \alpha , \quad a_1= a \alpha , \end{aligned}$$

95 $$\begin{aligned} b_1&= a(1-\alpha ) + (1+b), \quad b_2 = \alpha _2 + (1-\alpha ), \quad b_3= 1- \alpha , \end{aligned}$$

96 $$\begin{aligned} b_4&= \frac{b_1}{b_2}, \quad a_2=a_1-\alpha \frac{b_1}{b_2} \quad and \quad (\wp *\chi )(t)=\int _0^t \wp (t)\chi (t-\tau )d\tau . \end{aligned}$$

### Exact solution of fluid velocity by using CPC derivative operator

Solution of Eq. (14) with the application of Laplace integral transformation, then its solution in the following form

97 $$\begin{aligned} \left( a+(1+b)\left[ \frac{k_{1}(\alpha )}{s} + k_{0}(\alpha )\right] s^{\alpha }\right) \bar{u}(y,s)&=\left( 1+\alpha _2\left[ \frac{k_{1}(\alpha )}{s} + k_{0}(\alpha )\right] s^{\alpha }\right) \frac{d^{2}{\bar{u}(y,s)}}{d{y}^{2}} \nonumber \\&+G_{r}\bar{\theta }(y,s)+G_{m} \bar{C}(y,s), \end{aligned}$$

rearrange the above equation then it can be written as:

98 $$\begin{aligned} \frac{d^{2}{\bar{u}(y,s)}}{d{y}^{2}}-\left( \frac{a+d_1 A(s)}{1+ \alpha _2 A(s)}\right) \bar{u}(y,s)= - \left( \frac{1}{1+ \alpha _2 A(s)}\right) \left( G_{r}\bar{\theta }(y,s)+G_{m}{\bar{C}(y,s)}\right) , \end{aligned}$$

where $A(s)=\left[ \frac{k_{1}(\alpha )}{s} + k_{0}(\alpha )\right] s^{\alpha }$ and $d_1=1+b$ by using the Eqs. (26) and (53) in Eq. (98) then the solution in general form is represented as:

99 $$\begin{aligned} \bar{u}(y,s)&=c_{5}e^{y\sqrt{\frac{a+d_1 A(s)}{1+ \alpha _2 A(s)}}}+c_{6}e^{-y\sqrt{\frac{a+d_1 A(s)}{1+ \alpha _2 A(s)}}}-\left( \frac{G_{r}\left( \frac{1}{s}-\frac{a_0}{s+b_0}\right) e^{-y\sqrt{P_r A(s)}}}{P_r A(s)(1+ \alpha _2 A(s))-(a+ d_1 A(s)) }\right) \nonumber \\&-\left( \frac{G_{m}\left( \frac{1}{s}\right) e^{-y\sqrt{S_c A(s)+\lambda }}}{(S_c A(s)+\lambda )(1+ \alpha _2 A(s))-(a+ d_1 A(s)) }\right) , \end{aligned}$$

to determine unknowns $c_{5}$ and $c_{6}$, using ${\bar{u}(0,s)}=\frac{1}{s-i \epsilon }$ and ${\bar{u}(y,s)}\rightarrow 0$ as $y\rightarrow \infty$, we get

100 $$\begin{aligned} \bar{u}(y,s)&=\frac{1}{s-i \epsilon }e^{-y\sqrt{\frac{a+d_1 A(s)}{1+ \alpha _2 A(s)}}}+\left( \frac{G_{r}\left( \frac{1}{s}-\frac{a_0}{s+b_0}\right) }{P_r A(s)(1+ \alpha _2 A(s))-(a+ d_1 A(s)) }\right) \left[ e^{-y\sqrt{\frac{a+d_1 A(s)}{1+ \alpha _2 A(s)}}}-e^{-y\sqrt{P_r A(s)}} \right] \nonumber \\&+\left( \frac{G_{m}\left( \frac{1}{s}\right) }{(S_c A(s)+\lambda )(1+ \alpha _2 A(s))-(a+ d_1 A(s)) }\right) \left[ e^{-y\sqrt{\frac{a+d_1 A(s)}{1+ \alpha _2 A(s)}}}-e^{-y\sqrt{S_c A(s)+\lambda }} \right] . \end{aligned}$$

Similarly, we find the Laplace inverse of $\bar{u}(y,s)$, to obtain *u*(*y*, *t*), as we have computed for CF and ABC fractional derivative operators. The function $G_{h,b,l}(.,\tau )$ is known as G-function and is defined as $\frac{s^{b}}{(s^{h}-j)^{l}}=\mathcal {L}\left\{ G_{h,b,l}(j,\tau )\right\}$ with $Re(hl-b)>0$, $Re(s)>0$, $\left| \frac{j}{s^{h}} \right| <1$.

For validation purpose of the current study, we recovered the same velocity field equations Eq. (71) as Sami Ul Haq et al.^48^ investigated with exponential heating by removing the term *Gm*, i.e, $Gm=0$. Also, we obtained the same velocity field equations Eq. (84) as Ying-Qing Song et al.^49^ investigated with exponential heating by removing the term *Gm*, i.e, $Gm=0$. Further, in all of three cases CPC, CF and ABC, we get the classical case when $\alpha \rightarrow 1$.This prove the authenticity of our results.

## Results and discussion

**Figure 2:**
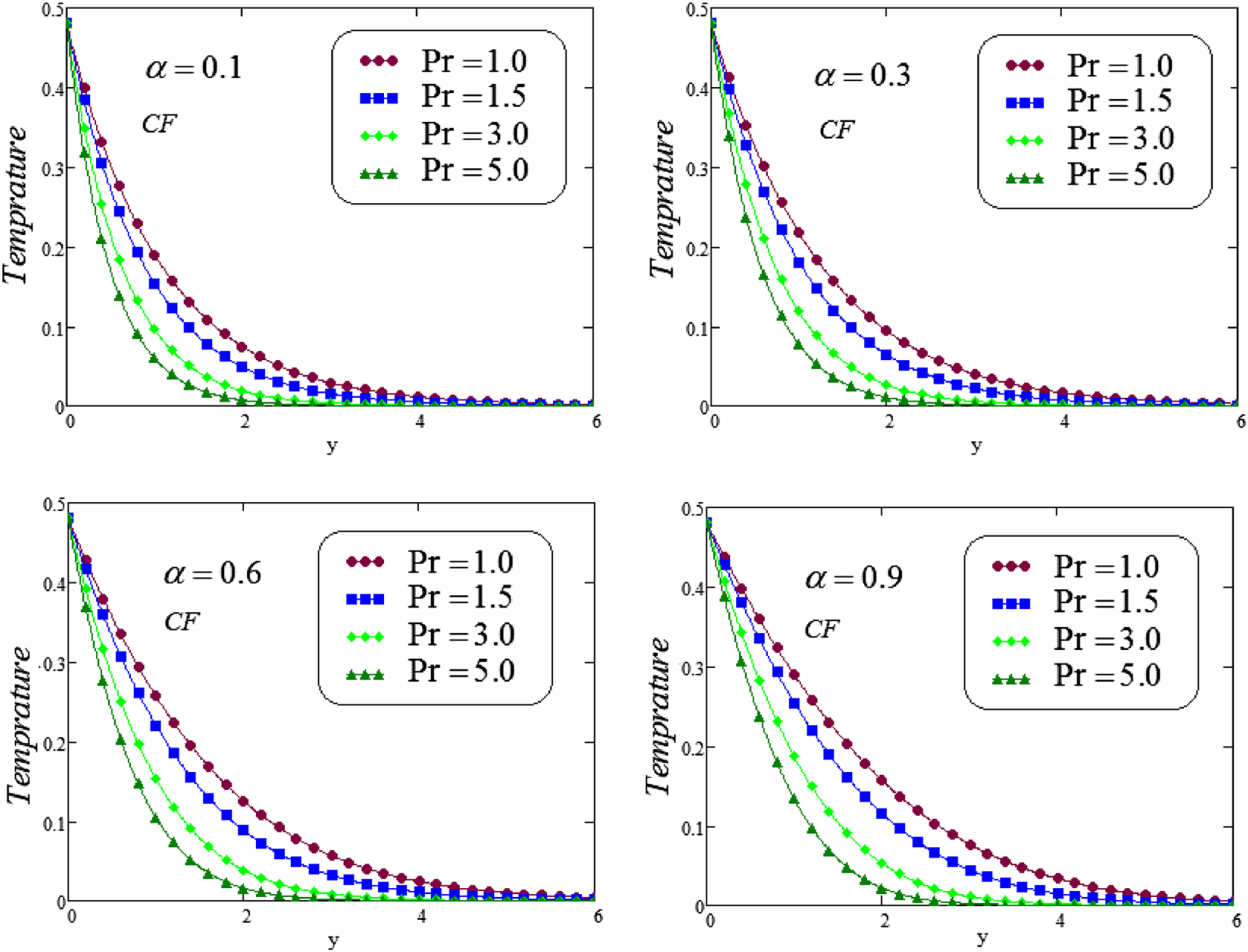
Simulation to illustrate the temperature profile for varying the values of *Pr* via CF at distinct values of the fractional parameter $\alpha$.

**Figure 3:**
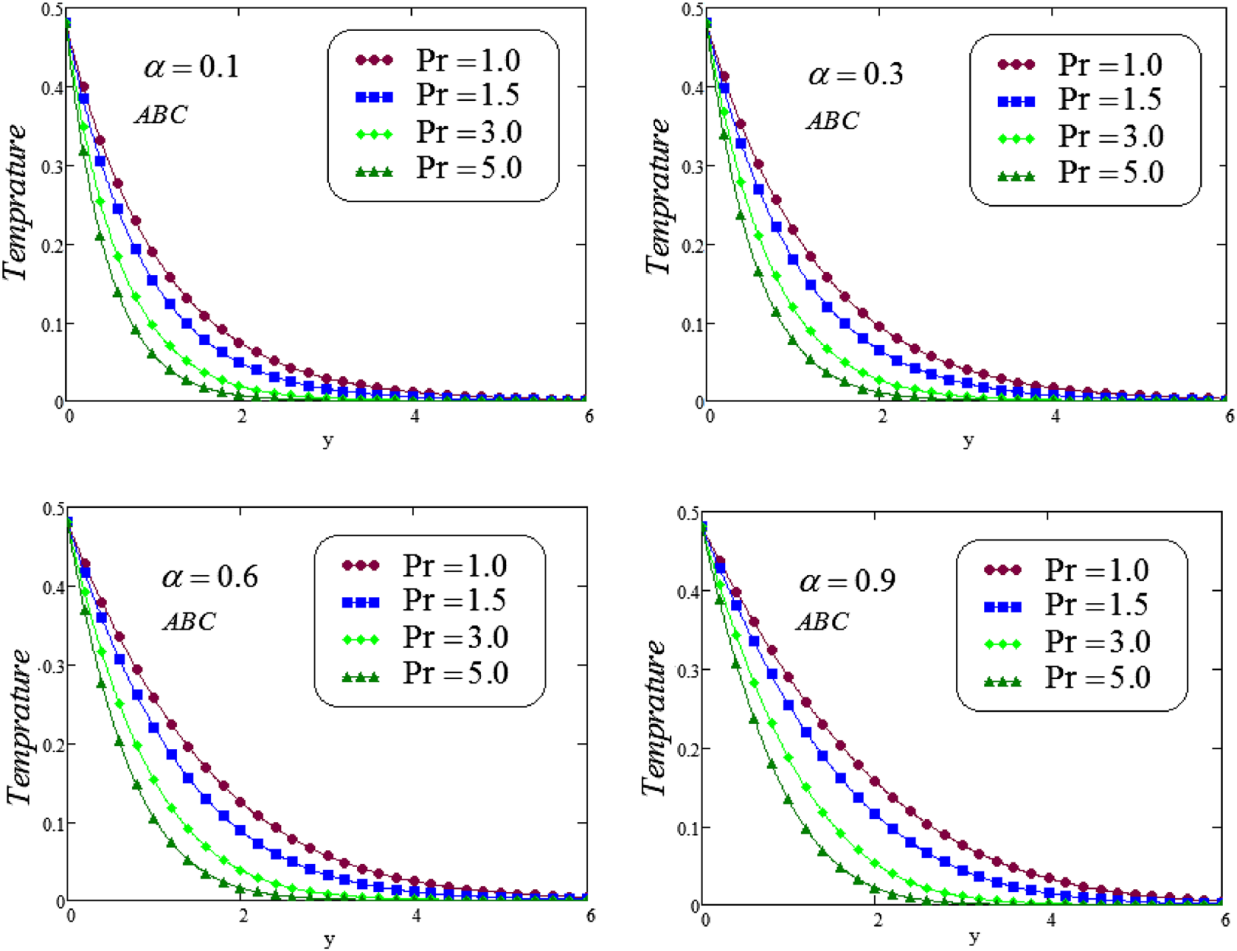
Simulation to illustrate the temperature profile for varying the values of *Pr* via ABC at distinct values of the fractional parameter $\alpha$.

**Figure 4:**
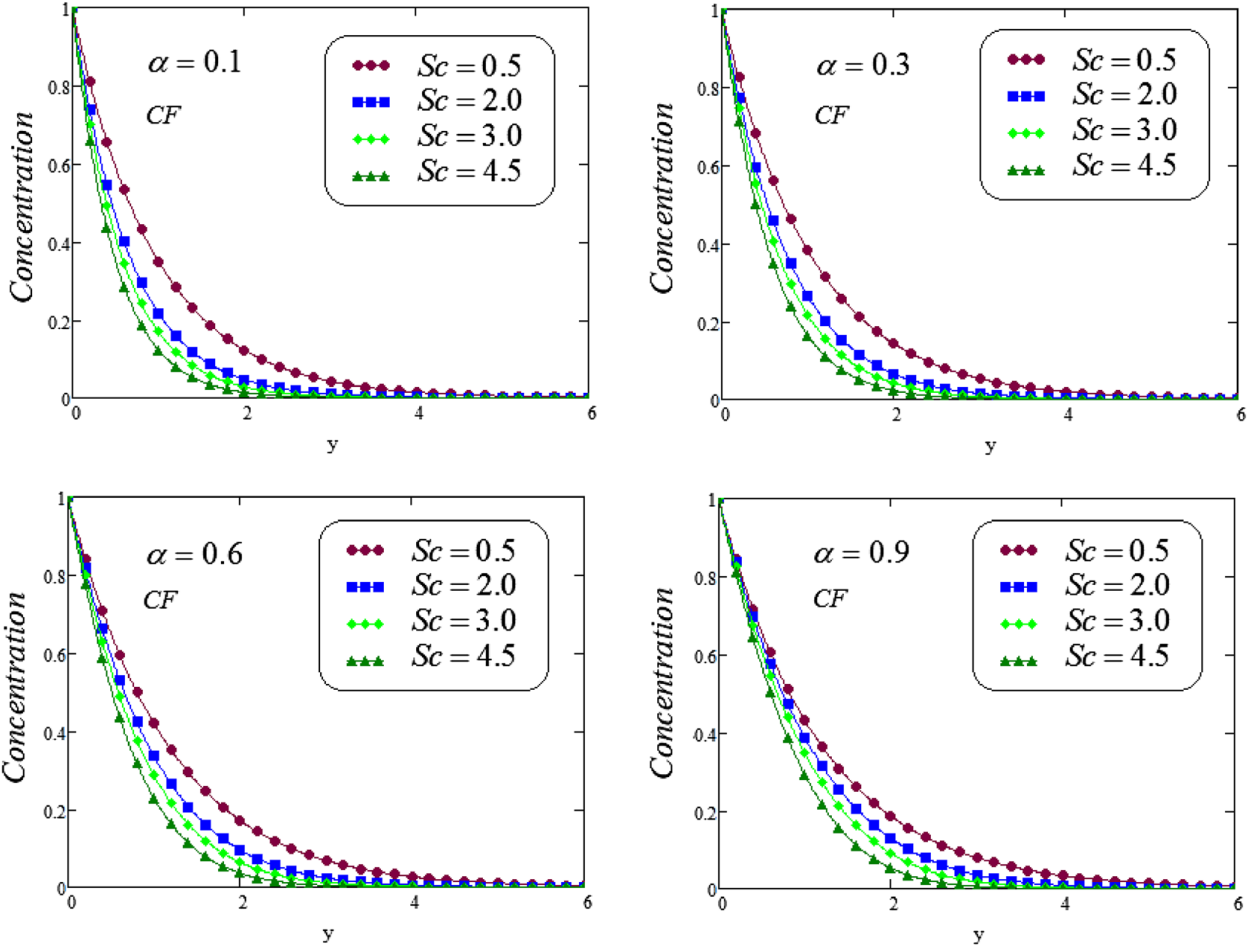
Representation of concentration profile via CF for distinct values of *Sc* at distinct values of the fractional parameter $\alpha$.

**Figure 5:**
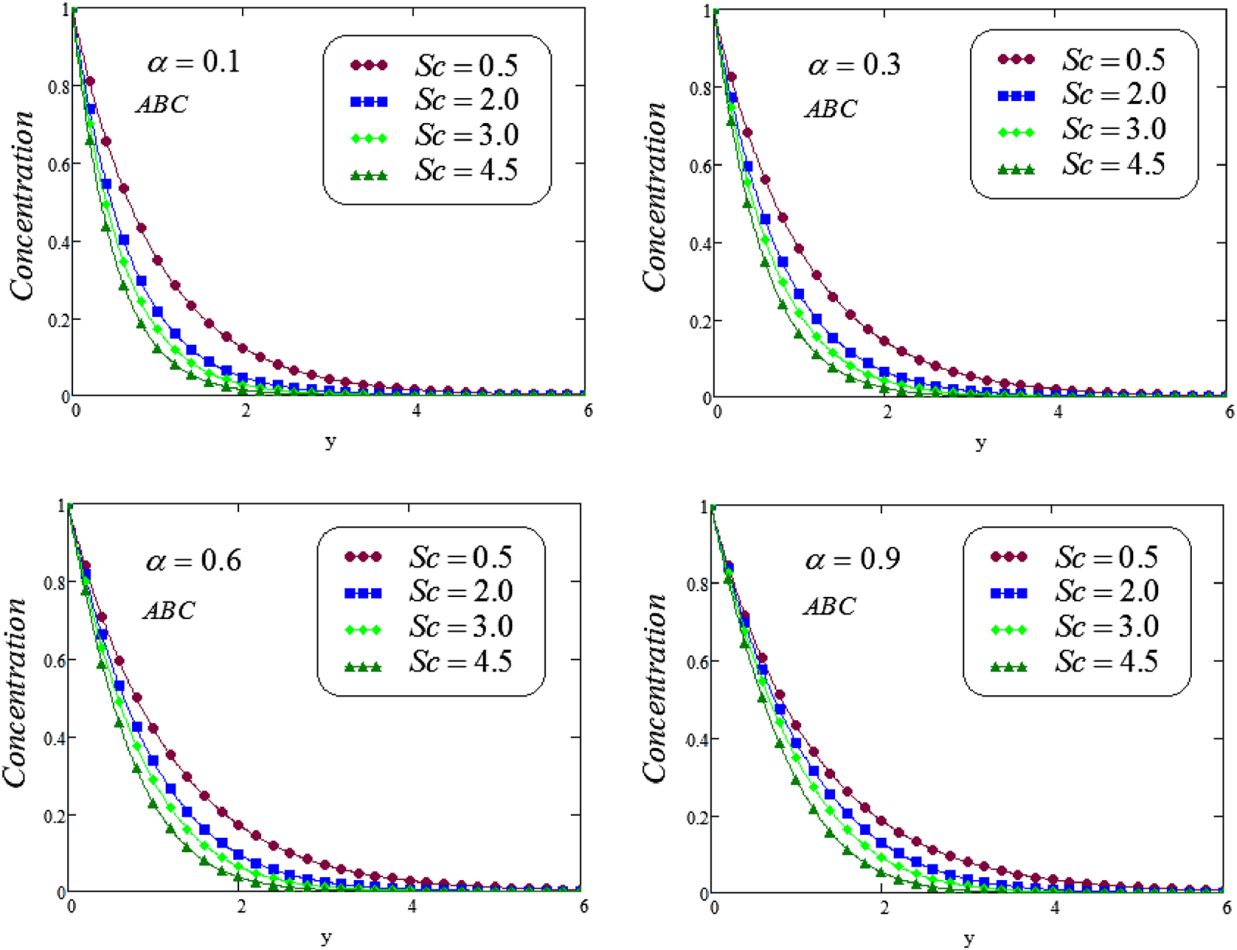
Representation of concentration profile via ABC for distinct values of *Sc* at distinct values of the fractional parameter $\alpha$.

**Figure 6:**
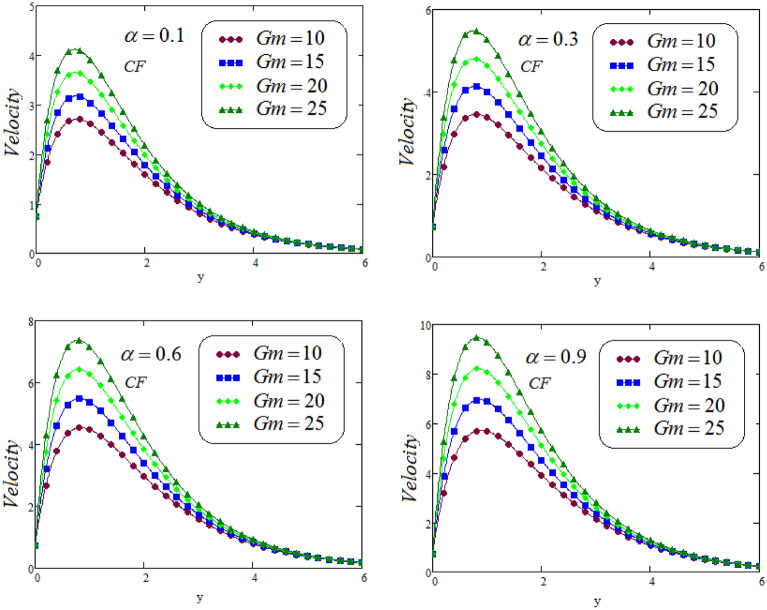
Representation of second grade fluid velocity via CF for distinct values of *Gm*.

**Figure 7:**
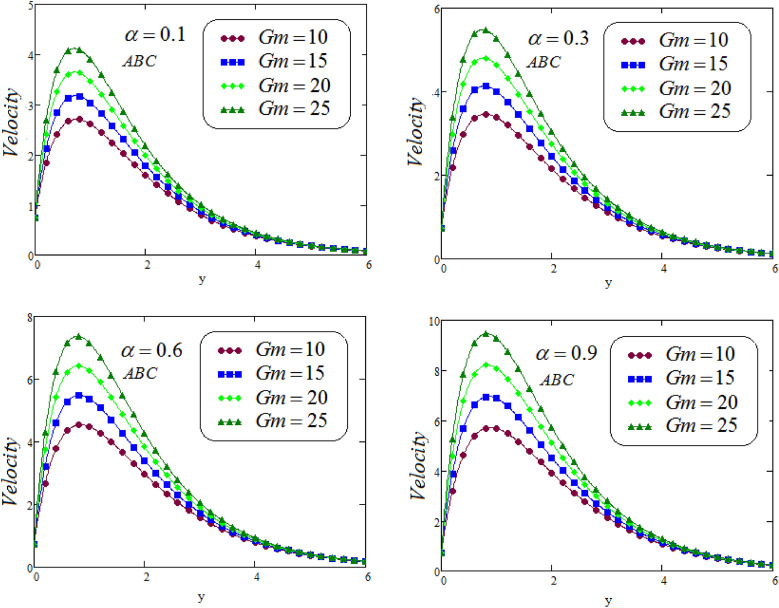
Representation of second grade fluid velocity via ABC for distinct values of *Gm*.

**Figure 8:**
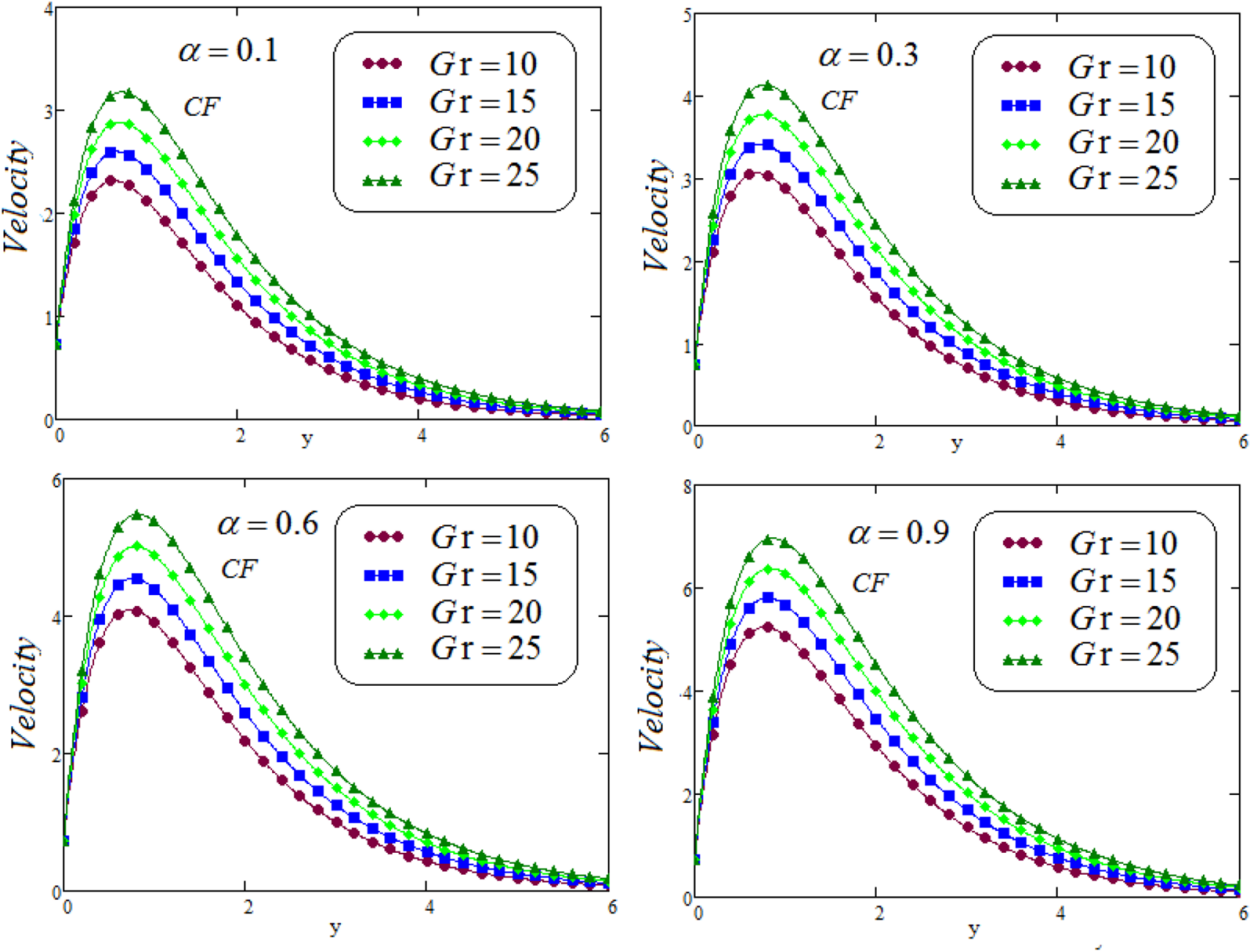
Representation of second grade fluid velocity via CF for distinct values of *Gr*.

**Figure 9:**
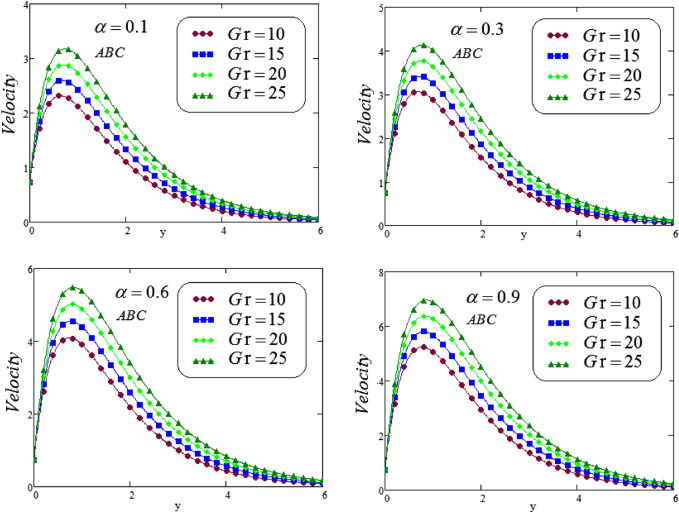
Representation of second grade fluid velocity via ABC for distinct values of *Gr* .

**Figure 10:**
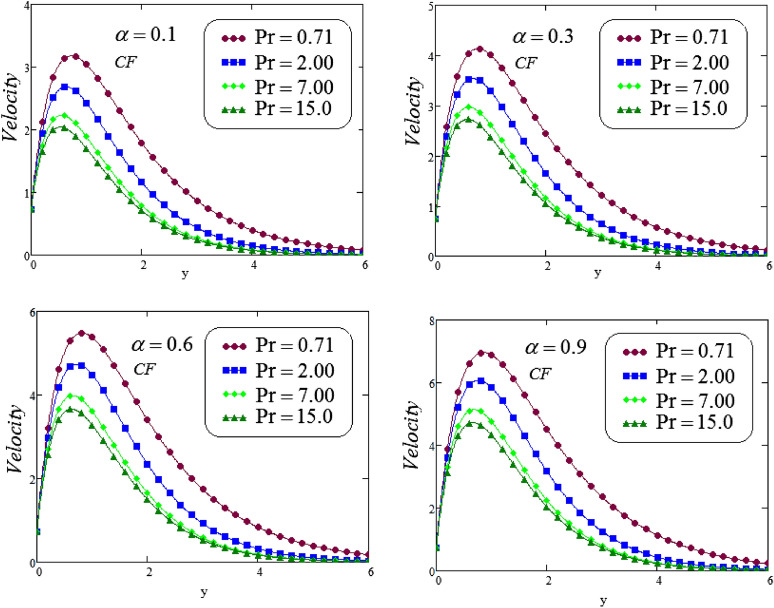
Representation of second grade fluid velocity via CF for distinct values of *Pr*.

**Figure 11:**
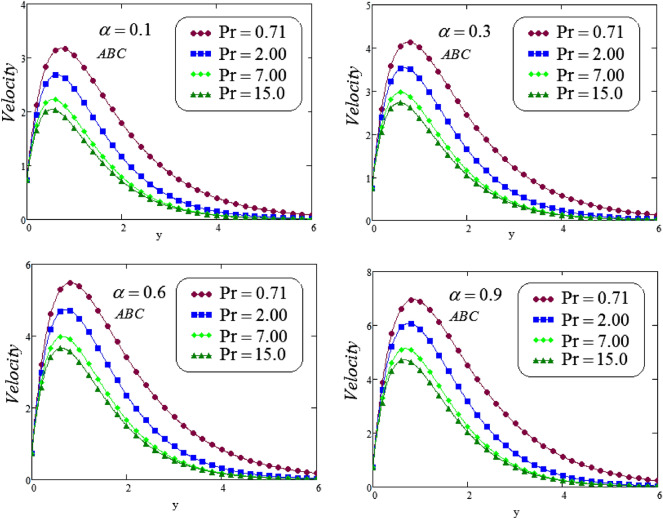
Representation of second grade fluid velocity via ABC for distinct values of *Pr*.

**Figure 12:**
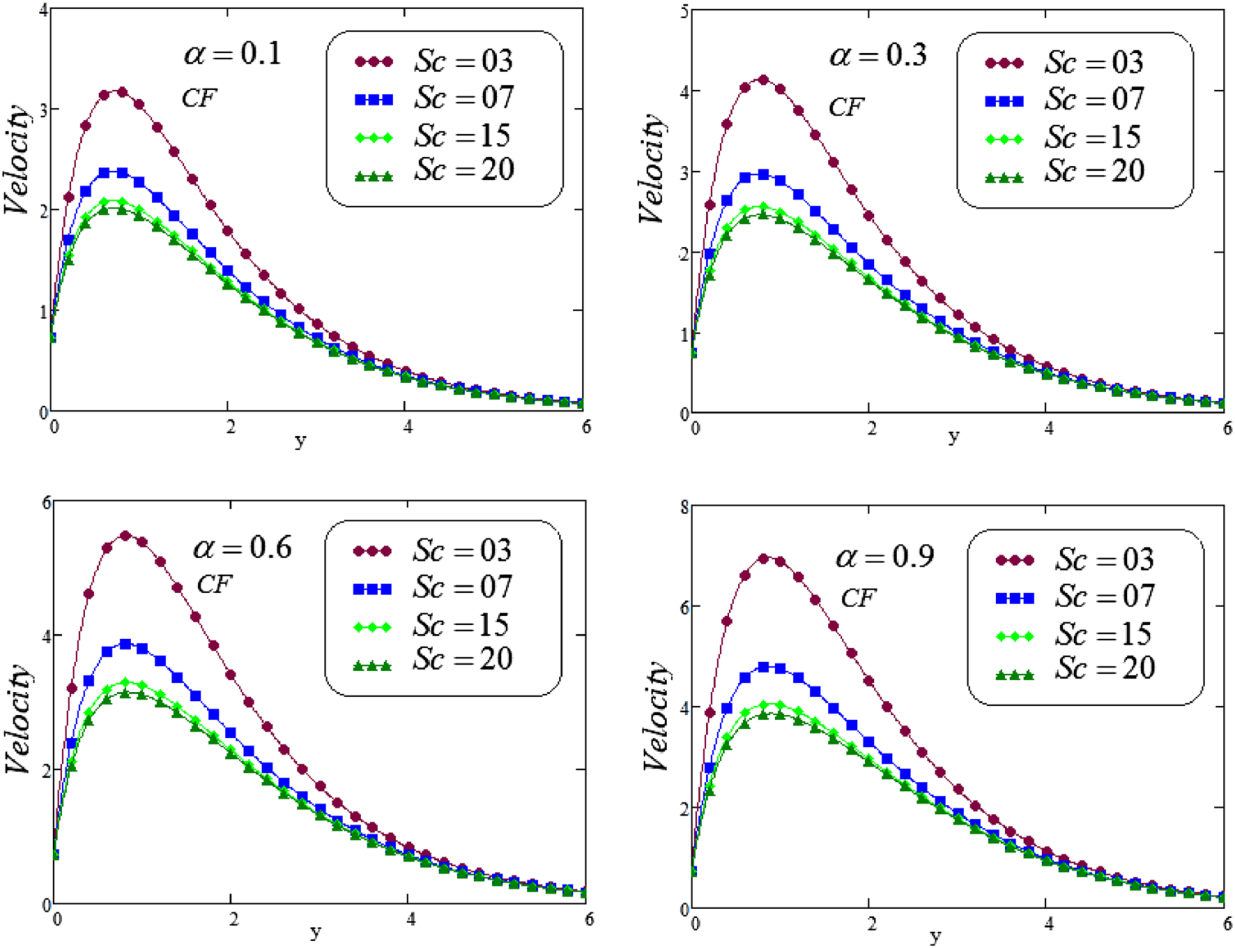
Representation of second grade fluid velocity via CF for distinct values of *Sc*.

**Figure 13:**
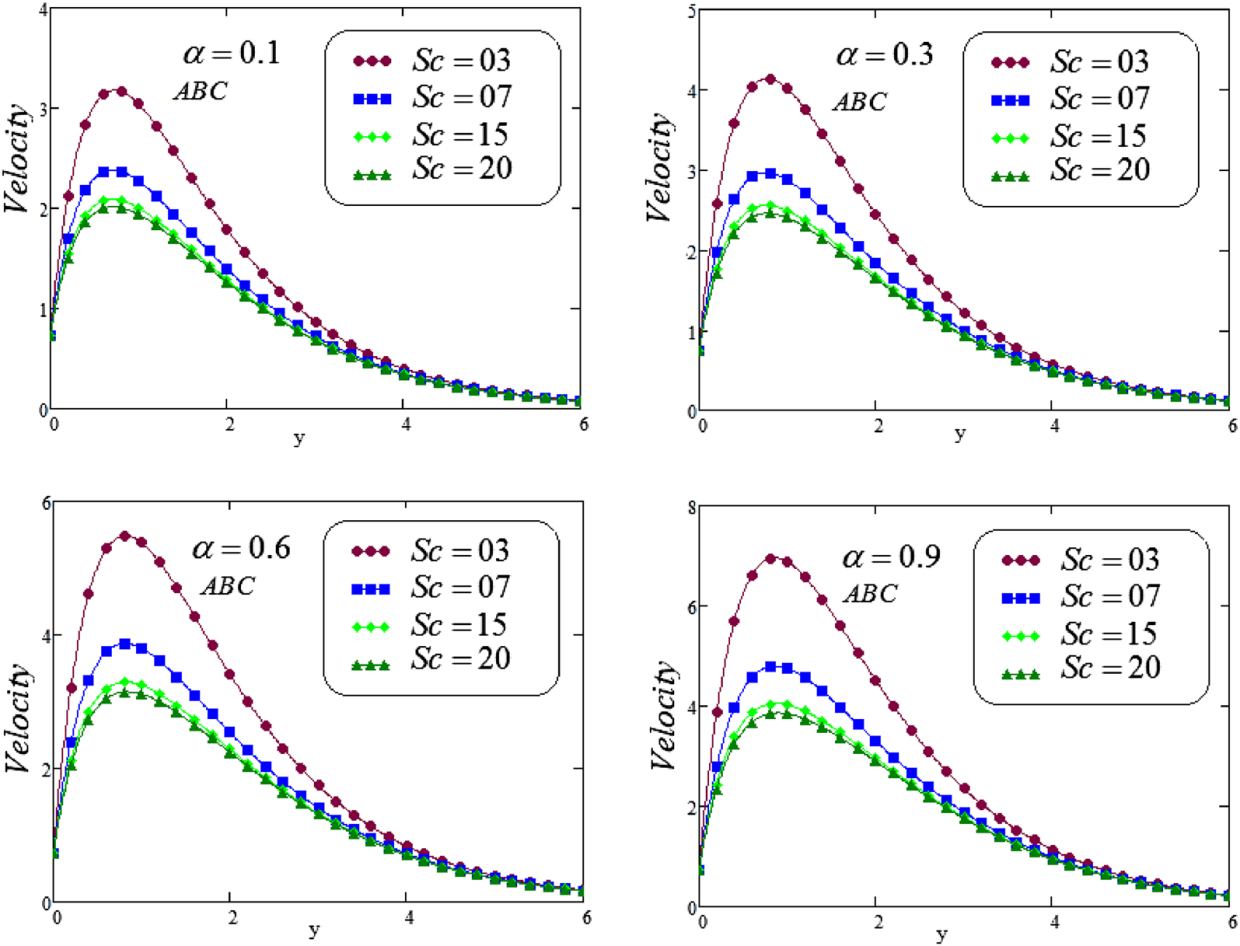
Representation of second grade fluid velocity via ABC for distinct values of *Sc*.

**Figure 14:**
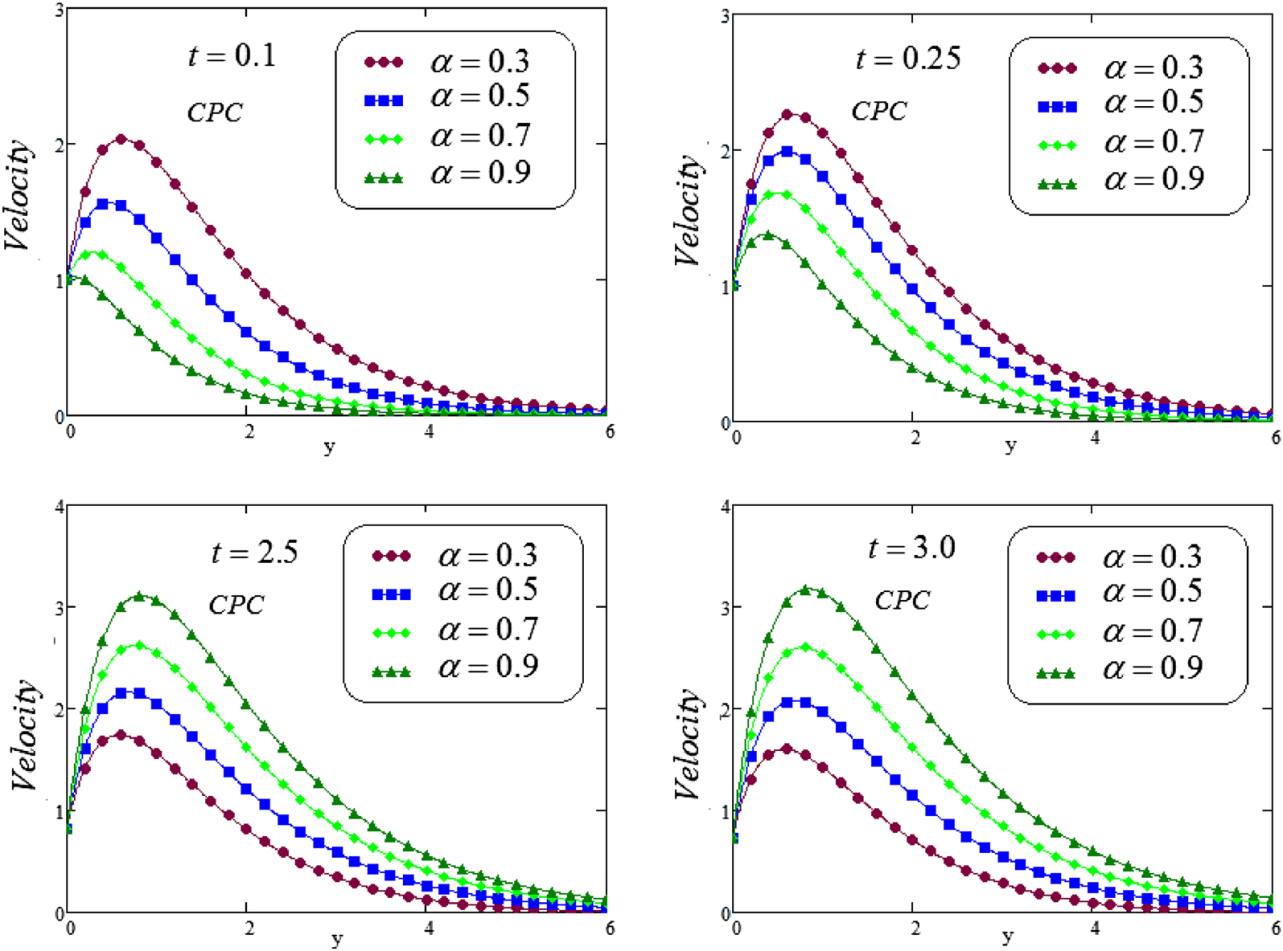
Trace of dimensionless velocity for CPC fractional operator for various values of $\alpha$.

**Figure 15:**
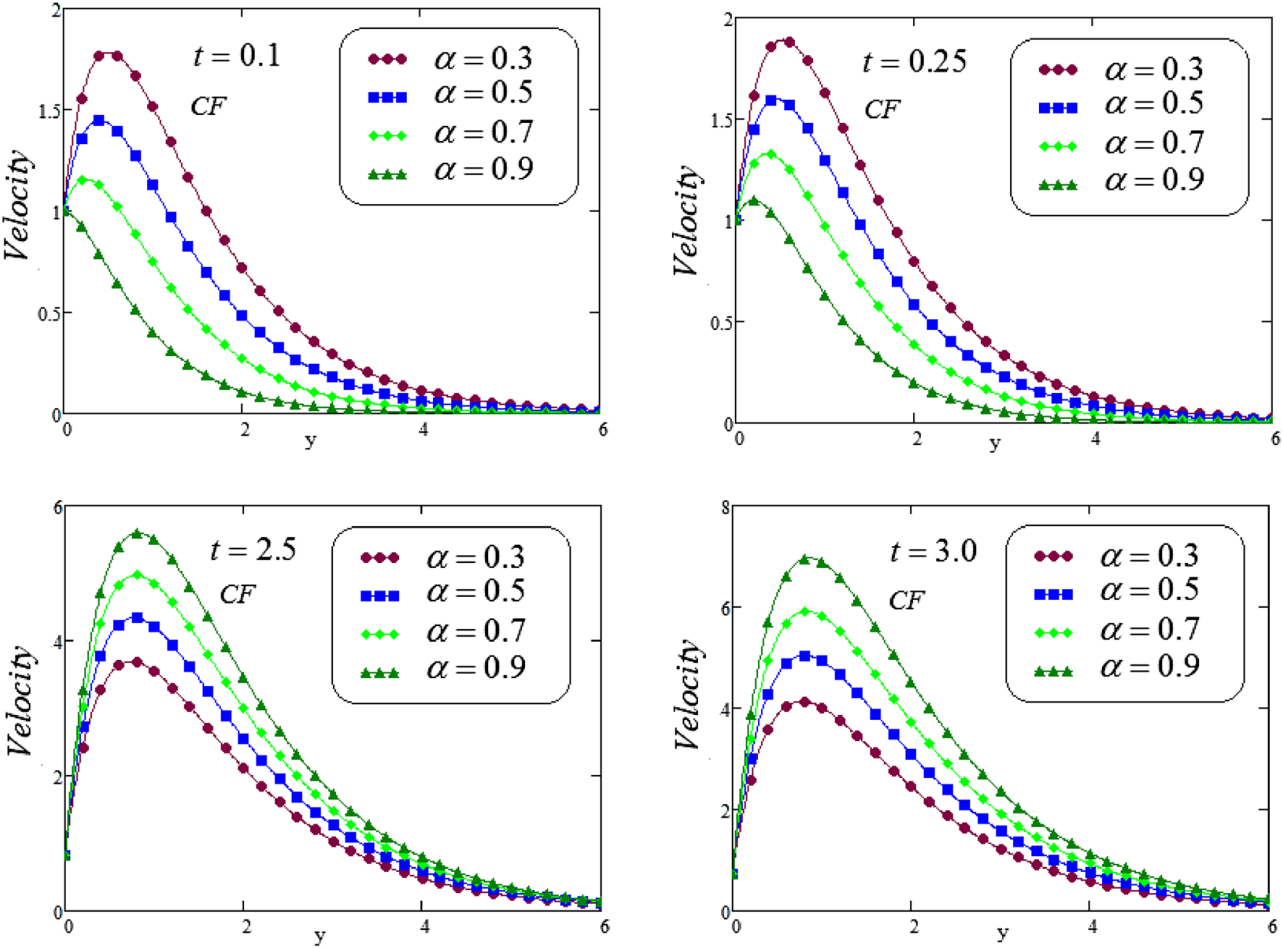
Trace of dimensionless velocity for CF fractional operator for varying the values of $\alpha$.

**Figure 16:**
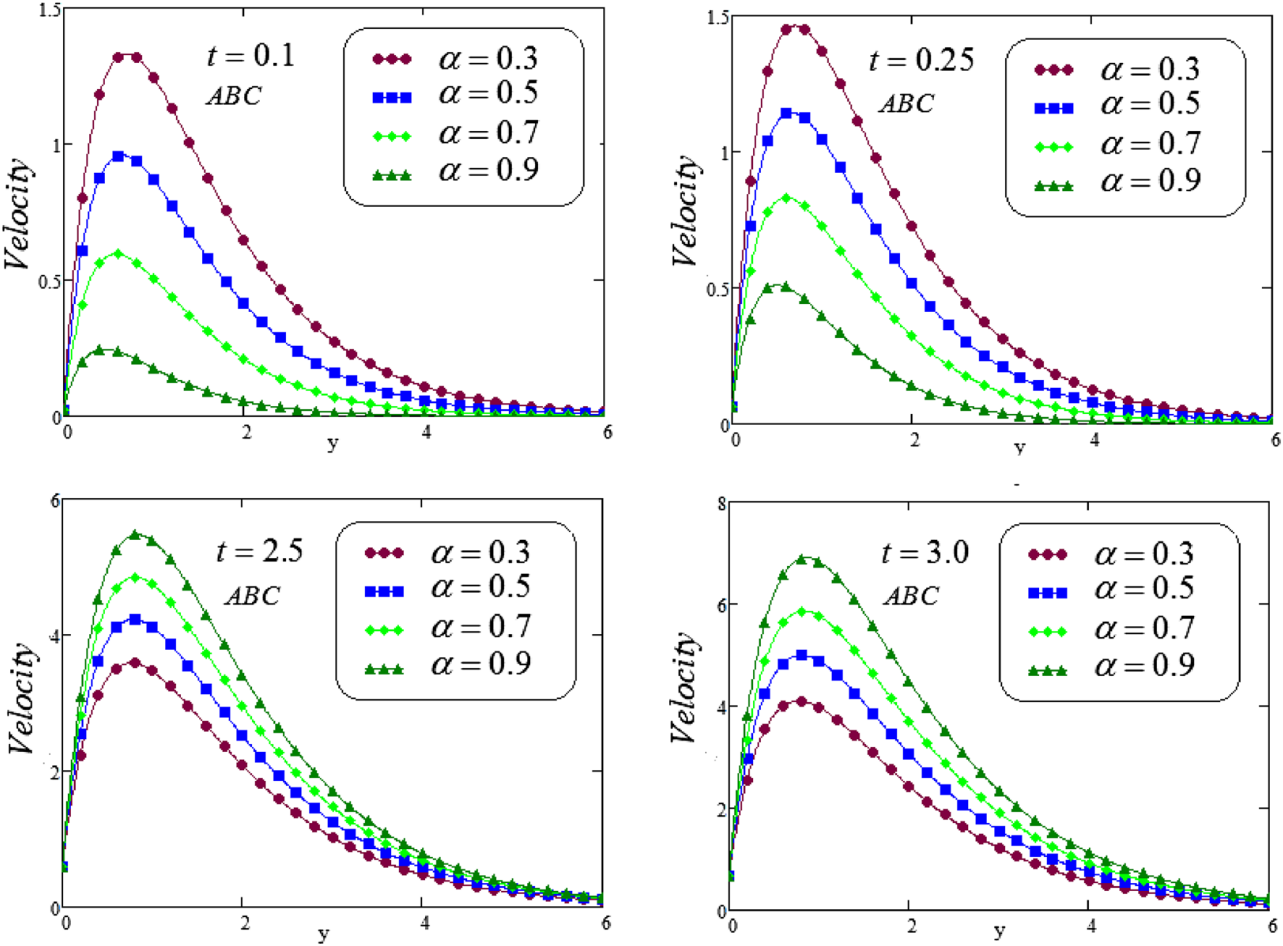
Trace of dimensionless velocity for ABC fractional operator for various values of $\alpha$.

**Figure 17:**
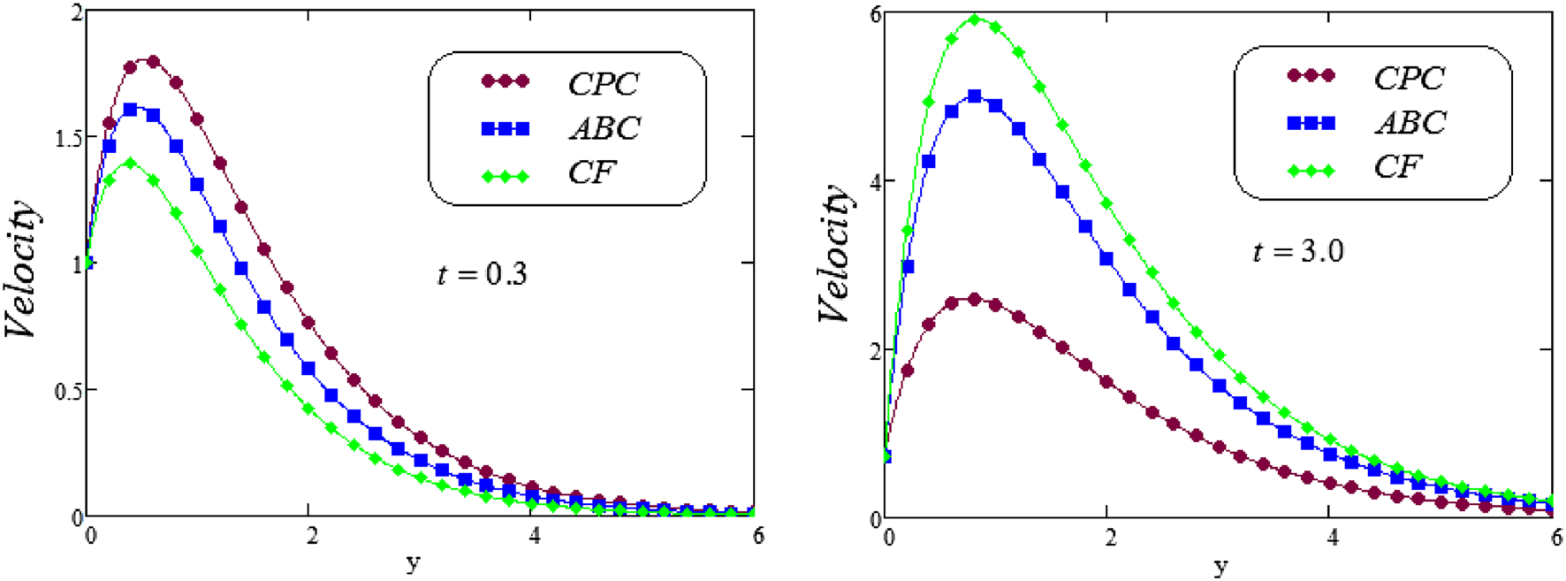
Trace of dimensionless velocity for comparison of CPC, ABC and CF models.

**Figure 18:**
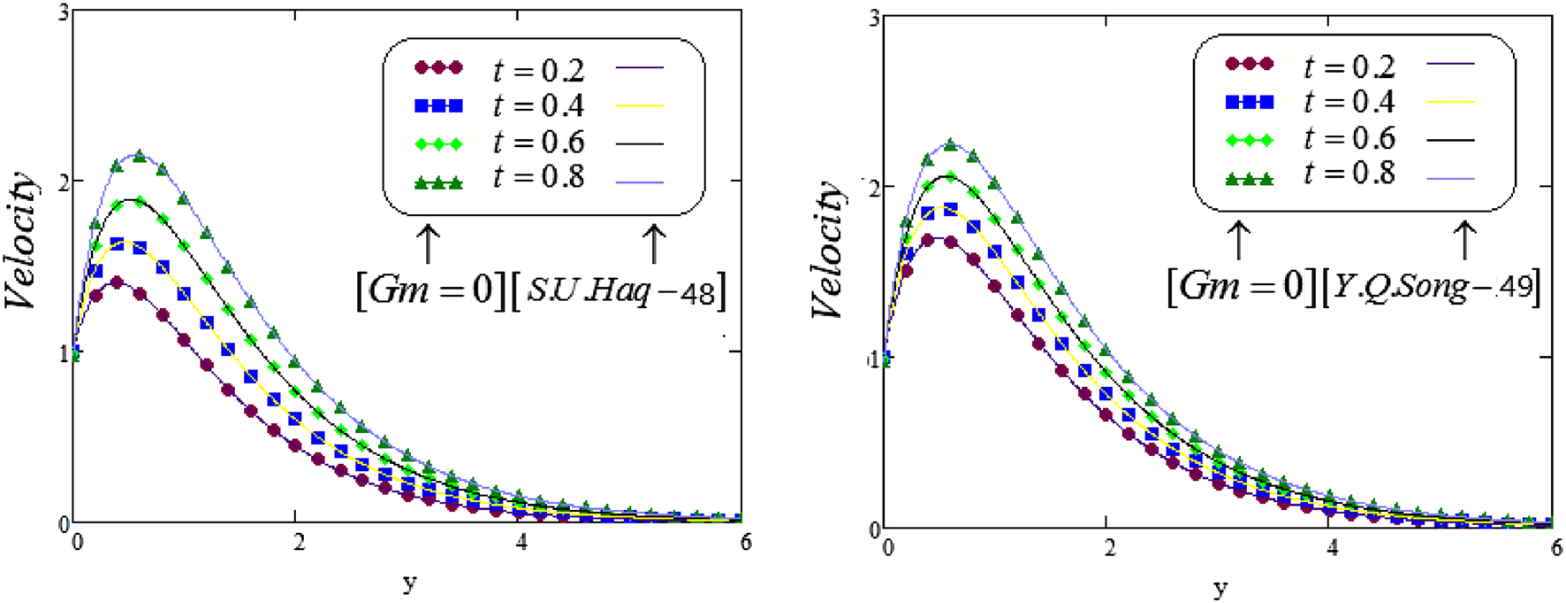
Comparison of the present velocity graphs, taking Gm = 0, with S. U. Haq and Y. Q. Song velocity graphs.

The unsteady second grade fluid with natural convective flow over flat plate of infinite length has been examined under exponential heating. Fractional model developed for non-dimensional velocity, concentration and energy equations by using CPC, CF and ABC fractional operators. Exact solution expressions obtained in terms of generalized G-function by simplifying the fractionalized model analytically with initial boundary conditions are provided for the proposed problem. For physical significance of various system parameters involved in the problem such as fractional parameter $\alpha$, thermal Grashof number *Gr*, Prandtl parameter *Pr*, Schmidt number *Sc* and mass Grashof number *Gm* on fluid velocity, concentration and temperature are evaluated and executed graphically in Figs. 2, 3, 4, 5, 6, 7, 8, 9, 10, 11, 12, 13, 14, 15, 16, 17 and 18 by using graphical Mathcad-Software, taking four values of fractional parameter $\alpha$ lies between 0 and 1, by considering $a_0=0.70$, $b_0=0.10$, $t=3$, $M=0.6$, $\lambda =0.7$ and $\alpha _2=0.5$

Figures 2 and 3 displays the Prandtl number $P_{r}$ effect on second grade fluid temperature distribution against *y* via CF and ABC operator, for different values of $P_{r}$, at four different values of fractional parameter $\alpha$. It is noticed that a decreasing effect on temperature in the boundary layer when the values of the Pradtl number enlarged. Physically, an increasing the values of Prandtl number that leads to an increases the fluid viscosity, because of this fluid becomes thicker due to viscosity increased , and as a result, fluid temperature decreased.

Figures 4 and 5 illustrates the behavior of *Sc* on concentration profile of second grade fluid by taking its dissimilar values of $\alpha = 0.1, 0.3, 0.6, 0.9$ via CF and ABC operator. From the curves it is analyzed that concentration profile reduced for large values of *Sc*, where the values of fractional parameters have been supposed between 0 and 1. Physically, boundary layer of concentration is declined due to change of Schmidt number from small to large.

Figures 6, 7, 8 and 9 portrays the influence of thermal Grashof numbers *Gr* and mass Grashof number *Gm* on second grade fluid flow against *y* via CF and ABC operator varying values of $\alpha = 0.1, 0.3, 0.6, 0.9$. Since *Gr* describes the fraction of thermal buoyancy force to viscous force, but *Gm* describes the fraction of species buoyancy force to viscous force, that are acting on the fluid transportation, as a result, with an increasing in *Gr* or *Gm* cause a remarkable increasing impact on the second grade fluid velocity. Physically, an increasing the values of thermal or mass Grashof numbers that leadsto decrease in viscous hydrodynamic forces, and as a result, the momentum of the second grade fluid is higher.

Figures 10 and 11, exhibits the effect of Prandl number $P_{r}$ on velocity corresponding to *y*, for different values of $P_{r}$, at four different values of fractional parameter $\alpha$ via CF and ABC operator. It is noticeable that a decreasing effect on velocity in the boundary layer when the values of the Prandtl number enlarged. Physically, an increasing the values of Prandtl number that causes to an increases the fluid viscosity, because of this fluid becomes thicker due to viscosity increased , and as a result, fluid velocity decreased.

Figures 12 and 13, the behavior of *Sc* on velocity curve is depicted, against *y*, for different values of *Sc*, at four different valuesof fractional parameter $\alpha$ via CF and ABC operator. It is noticed that a decreasing effect on concentration in the boundary layer when the values of the Schmidt number enlarged. Physically, the relative influence of of momentum diffusivity to species diffusivity is the definition of Schmidt number*Sc*. It is noticed that, momentum diffusivity is quicker than species diffusivity when Sc is greater than one $(Sc >1)$, but it is reverse when Sc is less than one $(Sc <1)$, and in case of $(Sc = 1)$, both species and momentum boundary layers have magnitude of the same order.

Figures 14, 15 and 16, illustrated the velocity profile for various values of $\alpha$ via a novel hybrid fractional operator CPC, CF and ABC with $t = 0.1, 0.25, 2.5, 3.0$, fluid velocity represents dual behavior for lesser and greater time, it can be seen that decline in velocity curve for smaller values of time, i.e, $t = 0.1, 0.25$ but reverse process is noticed for increasing time , i.e, for $t = 2.5, 3.0$ velocity curve is rising.

Figure 17, display the modification in velocity field, for comparative study among the three best fractional operators, namely a novel hybrid fractional operator CPC, CF and ABC. The graphical behavior and significant effect noticed on fluid velocity curve for $t = 0.3$ and $t = 3.0$ together with various parameters represents. The velocity graph is higher for CPC fractional operator as compared to CF and ABC, also the lowest velocity curves is observed for CF fractional operator for small time. It is noteworthy to point out that the graphical view of the fluid velocity, the ABC and CF fractional derivatives follow the velocity profile of CPC operator in respective order, but reverse order of operators observed for large time.

Figure 18, depicts the graphical behavior for comparative study, taking different values of *t*, and significant effect noticed on fluid velocity curve together with different parameters. It is noteworthy to point out that the graphical view of the fluid velocity is higher. Further, it is observed that the same velocity field equations Eq. (71) as Sami Ul Haq et al.^48^ investigated with exponential heating by removing the term *Gm*, i.e, $Gm=0$. Also, we obtained the same velocity field equations Eq. (84) as Ying-Qing Song et al.^49^ investigated with exponential heating by removing the term *Gm*, i.e, $Gm=0$.

## Conclusion

In this paper, unsteady second grade fluid flow under the effect of MHD along with exponential heating as well as Darcy’s law near an oscillating infinitely flat plate saturated in a permeable medium is analyzed. For the sake of better rheology of differential type fluid, developed a fractional model by employing the new definition of CPC, CF and ABC fractional derivative operators and exact solution obtained by using Laplace integral transformation. For several physical significance of various system parameters, the graphical representations of the analytical solutions illustrated the main results of the present work. Also, in the literature, it is observed that to derived analytical results from fractional fluid models developed by the various fractional operators, is difficult and this article contributing to answer the open problem of obtaining analytical solutions the fractionalized fluid models. Some essential major concluding observations obtained from the graphical analysis are summarized as follows:

- The temperature distribution corresponding to smaller and larger values of *Pr* has disclosed quick and thicker heat diffusivity;
- Mass concentration profile represented the decreasing behavior as the values of *Sc* increases, this is because of the relative thickness of the hydrodynamic layer.
- Increasing values of *Sc* that declines the velocity field.
- The growing values of *Gr* and *Gm* stimulates the velocity field.
- An increase in the values of *Pr* causes to decline velocity contour.
- It can be seen that fluid velocity graphs represents dual behavior for small and large time.
- It can be noticed that the second grade fluid velocity represents same behavior for small and large values of $\alpha$ via CPC, CF and ABC
- It is depicted that the fluid’s velocity for constant caputo-proportional hybrid derivative operator is greater than Atangana Baleanu and Caputo Fabrizio models respectively for small time but reverse order observed for large time.
- In comparison with Atangana Baleanu and Caputo Fabrizio fractional derivatives, the CPC fractional model is exceptionally suitable for simulating the history of velocity function.

## Data Availability

The data used in current study, available within the article that support the findings of the present research work.

## List of symbols

*u*: Non-dimensional velocity $\left( - \right)$
$$\mu$$: Dynamic viscosity $$\left( \text {kg} \; {{\text {m}}^{-1}}{{\text {s}}^{-1}} \right)$$
$\upsilon$: Kinematic coefficient of viscosity $\left( {{\text {m}}^{2}}{{\text {s}}^{-1}} \right)$
*G**r*: Thermal Grashof number $\left( - \right)$
*G**m*: Mass Grashof number $\left( - \right)$
*g*: Acceleration due to gravity $\left( \text {m} {{\text {s}}^{-2}} \right)$
$T_{w}$: Temperature of the plate $\left( \text {K} \right)$
$\beta _{T}$: Thermal expansion coefficient $\left( \text {K}^{-1} \right)$
$\beta _{C}$: Volumetric coefficient of concentration expansion $\left( \text {K}^{-1} \right)$
$T_{\infty }$: Temperature of fluid far away from the plat $\left( \text {K} \right)$
$$\rho$$: Fluid density $$\left( \text {kg}\; {{\text {m}}^{-3}} \right)$$
$C_{w}$: Concentration of the fluid near the plate $\left( \text {kg}\; {{\text {m}}^{-3}} \right)$
$C_{\infty }$: Concentration of the fluid far away from the plate $\left( \text {kg}\; {{\text {m}}^{-3}} \right)$
$$\sigma$$: Electrical conductivity $$\left( \text {s}\; {{\text {m}}^{-1}} \right)$$
$\alpha _2$: Second grade fluid parameter $\left( - \right)$
$C_{p}$: Specific heat at constant pressure $\left( \text {j}\; {{\text {kg}}^{-1}}{{\text {K}}^{-1}} \right)$
*P**r*: Prandtl number $$\left( - \right)$$
*S**c*: Schmidt number $$\left( - \right)$$
$$\delta _{m}$$: Mass diffusivity $$\left( {{\text {m}}^{2}}\; {{\text {s}}^{-1}} \right)$$
$$u_{0}$$: Characteristic velocity $$\left( {{\text {m}}}\; {{\text {s}}^{-1}} \right)$$
$$\theta$$: Dimensionless temperature $$\left( - \right)$$
*C*: Dimensionless concentration $$\left( - \right)$$
*M*: Total magnetic field $\left( - \right)$
*s*: Laplace transform parameter $\left( {\text {s}}^{-1} \right)$
$M_{0}$: Imposed magnetic field $\left( \text {W}\; {{\text {m}}^{-2}} \right)$
*t*: Time $$\left( \text {s} \right)$$
*k*: Thermal conductivity of the fluid $\left( \text {W}\; {{\text {m}}^{-2}}\;{{\text {K}}^{-1}} \right)$
*P*: Pressure $$\left( \text {N}\; \text {m}^{-2} \right)$$
$k_1$: Coefficient of Rosseland absorption $\left( - \right)$
$\sigma _1$: Stefan–Boltzmann constant $\left( \text {W}\;{{\text {m}}^{-2}}\;{{\text {K}}^{-4}} \right)$
*K*: Permeability of porous medium $\left( {\text {m}}^{2}\right)$
$$\alpha$$: Fractional parameter $$\left( - \right)$$
